# Stem Cell Determinant SOX9 Promotes Lineage Plasticity and Progression in Basal-like Breast Cancer

**DOI:** 10.1016/j.celrep.2020.107742

**Published:** 2020-06-09

**Authors:** John R. Christin, Chunhui Wang, Chi-Yeh Chung, Yu Liu, Christopher Dravis, Wei Tang, Maja H. Oktay, Geoffrey M. Wahl, Wenjun Guo

**Affiliations:** 1 Ruth L. and David S. Gottesman Institute for Stem Cell and Regenerative Medicine Research, Albert Einstein College of Medicine, Bronx, NY 10461, USA; 2 Department of Cell Biology, Albert Einstein College of Medicine, Bronx, NY 10461, USA; 3 Gene Expression Laboratory, Salk Institute for Biological Studies, La Jolla, CA 92037, USA; 4 Department of Breast Surgery, The First Affiliated Hospital of Guangzhou Medical University, Guangzhou 510120, China; 5 Department of Pathology, Albert Einstein College of Medicine/Montefiore Medical Center, Bronx, NY 10467, USA; 6 Albert Einstein Cancer Center, Albert Einstein College of Medicine, Bronx, NY 10461, USA; 7 Department of Anatomy and Structural Biology, Albert Einstein College of Medicine, Bronx, NY 10461, USA; 8 Gruss-Lipper Biophotonic Center, Albert Einstein College of Medicine, Bronx, NY 10461, USA; 9 Integrated Imaging Program, Albert Einstein College of Medicine, Bronx, NY 10461, USA; 10 Present address: Pfizer, Inc., San Diego, CA 92121, USA; 11 Present address: Janssen Pharmaceuticals, La Jolla, CA, 92121, USA; 12 These authors contributed equally; 13 Lead Contact

## Abstract

Lineage plasticity is important for the development of basal-like breast cancer (BLBC), an aggressive cancer subtype. While BLBC is likely to originate from luminal progenitor cells, it acquires substantial basal cell features and contains a heterogenous collection of cells exhibiting basal, luminal, and hybrid phenotypes. Why luminal progenitors are prone to BLBC transformation and what drives luminal-to-basal reprogramming remain unclear. Here, we show that the transcription factor SOX9 acts as a determinant for estrogen-receptor-negative (ER^−^) luminal stem/progenitor cells (LSPCs). SOX9 controls LSPC activity in part by activating both canonical and non-canonical nuclear factor κB (NF-κB) signaling. Inactivation of TP53 and RB via expression of SV40 TAg in a BLBC mouse tumor model leads to upregulation of SOX9, which drives luminal-to-basal reprogramming *in vivo*. Furthermore, SOX9 deletion inhibits the progression of ductal carcinoma *in situ* (DCIS)-like lesions to invasive carcinoma. These data show that ER^−^ LSPC determinant SOX9 acts as a lineage plasticity driver for BLBC progression.

## In Brief

Basal-like breast cancer (BLBC) preferentially originates from ER-negative luminal stem/progenitor cells (LSPCs). Christin et al. show that the transcription factor SOX9 acts as a key regulator for these LSPCs. Inactivation of BLBC tumor suppressors co-opt SOX9 upregulation to promote luminal-basal reprogramming and tumor progression.

## Graphical Abstract

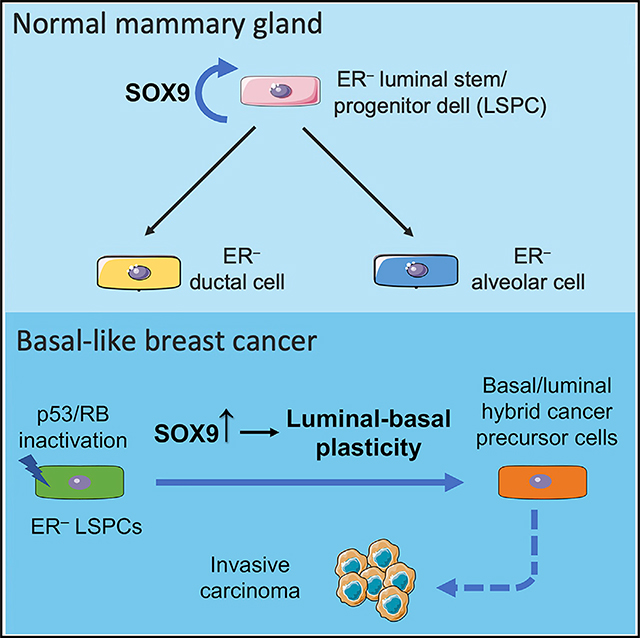

## INTRODUCTION

Lineage plasticity, the ability of committed cells to change cell states through dedifferentiation or transdifferentiation, is an important mechanism for tissue repair (Ge and Fuchs, 2018; Rajagopal and Stanger, 2016; Wahl and Spike, 2017). Cancer can co-opt this normal repair program to promote its initiation and progression (Ge and Fuchs, 2018; Le Magnen et al., 2018). Un-like normal tissues, where lineage plasticity is a transient state during tissue repair, cancer cells exhibit persistent plasticity. Various oncogenic mutations can enable cells to break down normal lineage restriction and acquire aberrant lineage potential (Ge et al., 2017; Van Keymeulen et al., 2015; Kopp et al., 2012; Koren et al., 2015). In addition, cancer cell plasticity can be perpetuated by an inflammatory tumor microenvironment (Ge and Fuchs, 2018; Le Magnen et al., 2018). This excess cellular plasticity is a major contributor to tumor heterogeneity (Wahl and Spike, 2017). Lineage plasticity has also been recognized as an important mechanism of drug resistance, allowing cancer cells to change cell states and escape from lineage-directed therapy (Ku et al., 2017; Mu et al., 2017; Zou et al., 2017). A better understanding of the underlying mechanisms driving lineage plasticity is important for developing more effective cancer therapy.

Basal-like breast cancer (BLBC), which includes the majority of triple-negative breast cancer, is an aggressive cancer subtype demonstrating high degrees of cellular plasticity (Prat and Perou, 2011; Wahl and Spike, 2017). Despite its prominent basal cell features compared to other breast cancer subtypes, BLBC is likely to originate from luminal progenitors (Lim et al., 2009, 2010; Molyneux et al., 2010; Proia et al., 2011). The global gene expression signature of BLBC is closely related to adult luminal progenitors and fetal mammary stem cells (Lim et al., 2009, 2010; Spike et al., 2012; Giraddi et al., 2018). Furthermore, transformation of luminal cells, but not basal cells, generates tumors resembling human BLBC (Keller et al., 2012; Molyneux et al., 2010). Interestingly, inactivation of the BLBC tumor suppressor BRCA1 or p53 leads to expansion of luminal progenitors in human patients and elicits a luminal-to-basal/mesenchymal transition in mouse models (Lim et al., 2009; Rios et al., 2019; Sau et al., 2016; Tao et al., 2017; Wang et al., 2019). Why certain luminal cells are predisposed to transformation by loss of BLBC tumor suppressors remains unclear, as do the mechanisms mediating the luminal-to-basal reprogramming. Addressing these questions would provide much needed clarity on the mechanisms of cell-state switching in breast cancer.

The mammary luminal epithelium is composed of estrogen-receptor-negative (ER^−^) and ER-positive (ER^+^) cells. We and others have shown that ER^−^ and ER^+^ luminal cells are two independent lineages that can be maintained by distinct stem/progenitor cells in the postnatal mouse mammary gland (Giraddi et al., 2015; Van Keymeulen et al., 2017; Rodilla et al., 2015; Wang et al., 2017). A population of SOX9^+^/NOTCH1^+^ cells maintain the self-renewal and regeneration of the ER^−^ lineage (Van Keymeulen et al., 2017; Rodilla et al., 2015; Wang et al., 2017). These cells overlap with the cell population previously considered as the origin of BLBC (Lim et al., 2009; Molyneux et al., 2010). SOX9 is a key developmental transcription factor that regulates the function of stem/progenitor cells in several epithelial tissues, including a role in inducing gland-reconstituting multipotent mammary stem cell activity (Guo et al., 2012; Jo et al., 2014). A recent *in vitro* study suggests that SOX9 plays a role in luminal progenitors (Domenici et al., 2019). Earlier work also showed *Sox9* deletion causes a transient delay in mammary ductal tree development (Malhotra et al., 2014). However, the function of SOX9 in LSPCs *in vivo* remains unclear. More importantly, the role of SOX9 in BLBC initiation and progression has not been studied. Here, we investigated the functional role of SOX9 in ER^−^ luminal stem/progenitor cells (LSPCs) and BLBC oncogenesis using *in vivo* genetic models.

## RESULTS

### SOX9 Controls ER^−^ Luminal Stem/Progenitor Activity

**Figure 1.**
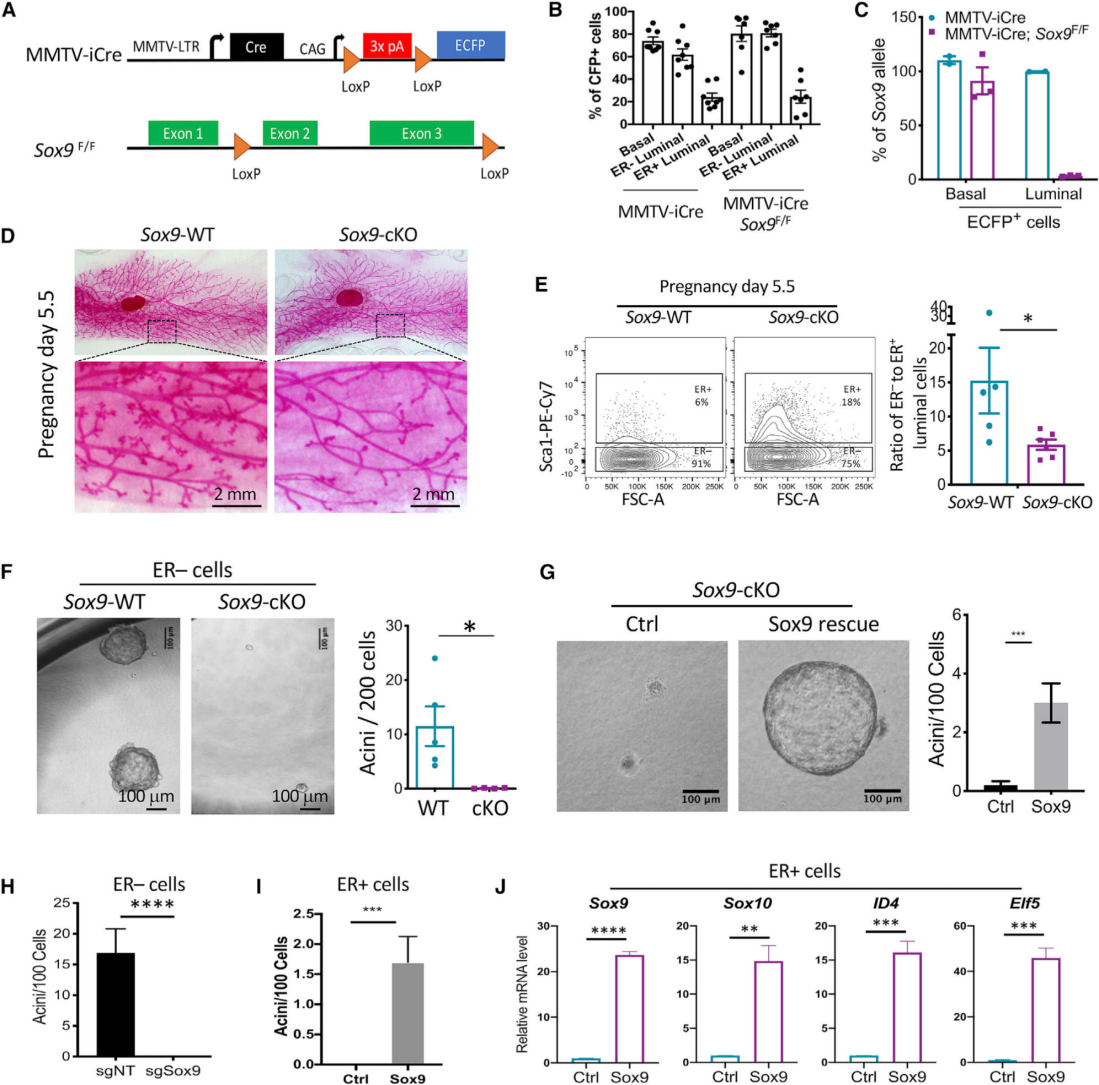
*Sox9* Deletion *In Vivo* Inhibits ER^−^ Luminal Stem/Progenitor Activity (A) Schematic of mouse models. (B) MMTV-iCre activity in mammary epithelial cell subpopulations, based on the ECFP reporter. Mean ± SEM (n = 7–8 per group). (C) Sox9 allele frequency in ECFP+ basal or luminal cells of indicated animals. Genomic DNA of fluorescence-activated cell sorting (FACS)-sorted cells was analyzed by qPCR. Mean ± SEM (n = 2–3 per group). (D) Representative whole-mount carmine stain of pregnancy day 5.5 mammary glands of *Sox9*-WT (*Sox9* ^F/F^,n= 6) or *Sox9*-cKO (MMTV-iCre; *Sox9* ^F/F^,n= 8) mice. Mean ± SEM. (E) Ratio of ER^−^ to ER^+^ luminal cells at pregnancy day 5.5 as determined by flow cytometry. Mean ± SEM (n = 5–6 per group). *p < 0.05. (F) Acinus-forming ability of ECFP+ ER− luminal cells sorted from *Sox9*-WT (MMTV-iCre) or *Sox9*-cKO (MMTV-iCre; *Sox9*^F/F^) mice at 2–3 months. Mean ± SEM (n = 4–5 per group). *p < 0.05. (G) Acinus-forming ability of ECFP^+^ ER^−^ luminal cells from MMTV-iCre; Sox9^F/F^ mice transduced with the Sox9-expressing or control lentiviral vector. Freshly FACS-sorted cells were transduced in organoid culture, puromycin selected, and then reseeded for measuring acinus-forming ability. Representative results of two experiments are shown. Mean ± SEM ***p < 0.001. (H) Acinus-forming capability of *Sox9-*WT ER- luminal cells transduced with the indicated sgRNA lentiviral vectors. Cells were transduced in organoid culture, selected with puromycin, and then re-seeded for measuring organoid-forming ability. Representative results of three experiments are shown. Mean ± SEM ****p < 0.0001. I) Acinus-forming ability of *Sox9*-WT ER^+^ luminal cells transduced with the Sox9 or control lentiviral vector. Cells were transduced in adherent culture, selected with puromycin, and then reseeded in organoid culture 3 days post-transduction. Representative results of two experiments are shown. Mean ± SEM. ***p < 0.001. (J) Expression levels of ER^−^ luminal transcription factors in ER^+^ luminal cells transduced by the Sox9-expressing or control vector as done in (I). Cells were analyzed by qRT-PCR 3 days post-transduction. Representative results of two experiments are shown. Mean ± SEM. **p < 0.01, ***p < 0.001, ****p < 0.0001.

Consistent with previous studies (Domenici et al., 2019; Wang et al., 2017), we found that ER^−^ luminal cells expressed markedly higher levels of *Sox9* mRNA relative to ER^+^ cells (Figures S1A and S1B). To determine the role of SOX9 in the mammary gland *in vivo*, we crossed mice carrying a floxed *Sox9* allele (*Sox9* ^F/F^) (Akiyama et al., 2002) to MMTV-iCre (codon-improved Cre) transgenic mice (Roussos et al., 2010). The MMTV-iCre transgene also contained a Cre-activatable ECFP reporter, which facilitates the identification of Cre-recombined cells (Figure 1A). In keeping with the known incomplete penetrance of the MMTV promoter in mammary epithelial cells (Hennighausen et al., 1995), MMTV-iCre was expressed in 60%–80% of basal and ER^−^ luminal cells but only ~20% of ER^+^ cells, based on the ECFP Cre reporter (Figure 1B). Similar results were found by directly measuring *Sox9* deletion in mammary subpopulations (Figure S1C). We further measured *Sox9* deletion in sorted ECFP^+^ cells. While the floxed *Sox9* allele was efficiently deleted in ECFP^+^ luminal cells, surprisingly, it was not deleted in ECFP^+^ basal cells (Figure 1C). The exact cause of lack of *Sox9* deletion in Cre-reporter-positive basal cells remains to be determined. However, efficient *Sox9* deletion in ECFP^+^ luminal cells in MMTV-iCre; *Sox9* ^F/F^ (referred to as *Sox9*-cKO henceforth) mice allowed us to investigate SOX9 function in the ER^−^ luminal lineage *in vivo*.

Nulliparous *Sox9*-cKO mice showed normal mammary ductal tree development (Figures S1D and S1E). However, there was a noticeable alveologenesis defect during early pregnancy in *Sox9-*cKO mice (Figure 1D) and, correspondingly, a 3-fold decrease in the ER^−^ to ER^+^ luminal cell ratio relative to control animals (Figure 1E). This is consistent with previous finding that SOX9^+^ LSPCs contribute to ER^−^ alveolar cells during pregnancy (Wang et al., 2017). The alveologenesis defect in *Sox9-*cKO was no longer present later in pregnancy (Figure S1D). One cause of the transient developmental phenotype in *Sox9*-cKO mice could be the incomplete deletion of *Sox9* in the ER− luminal lineage (Figures 1B and S1C).

To directly assess ER^−^ LSPC activity, we utilized a Matrigel-based organoid culture assay that specifically measures ER^−^ LSPC activity. This culture condition enables ER^−^ luminal cells to robustly generate acinar structures, with minimal growth from ER^+^ cells (Figure S1F). The *ex vivo* acinar structures maintained high levels of *Sox9* similar to those of the ER^−^ cells *in vivo* (Figure S1G). Compared to the control ER^−^ cells, the *Sox9* null ER^−^ cells were depleted of LSPC activity (Figure 1F). The defect of ER^−^ LSPCs in *Sox9*-cKO animals could be due to a cell-autonomous role of SOX9 in maintaining LSPC activity or a requirement of SOX9 in the development of LSPCs. Therefore, we re-expressed *Sox9* in *Sox9*-cKO cells *ex vivo* and found it rescued the LSPC activity (Figure 1G). Furthermore, acute deletion of *Sox9* in freshly sorted wild-type (WT) ER^−^ luminal cells by CRISPR greatly diminished LSPC activity *ex vivo* (Figures 1H and S1H). These results suggest a cell-autonomous, persistent requirement for SOX9 expression in maintaining LSPC activity.

To further test whether SOX9 is capable of inducing the ER^−^ LSPC state, we found that SOX9 ectopic expression could induce acinus-forming ability in ER^+^ cells to a level equivalent to 15% of the activity in endogenous ER^−^ cells (Figure 1I). Furthermore, SOX9 ectopic expression markedly increased the expression of multiple ER^−^ luminal cell signature genes, including *Sox10*, *Id4*, and *Elf5* (Figure 1J; Dravis et al., 2015; Lim et al., 2010). Interestingly, our previous study showed upregulation of SOX10 is required for induction of organoid-forming cells by SOX9 and SLUG (Guo et al., 2012). Together, the above results demonstrate that SOX9 is an important determinant of ER^−^ LSPC activity.

### SOX9 Upregulates Both Canonical and Non-canonical NF-κB Signaling in ER^−^ Luminal Cells

**Figure 2.**
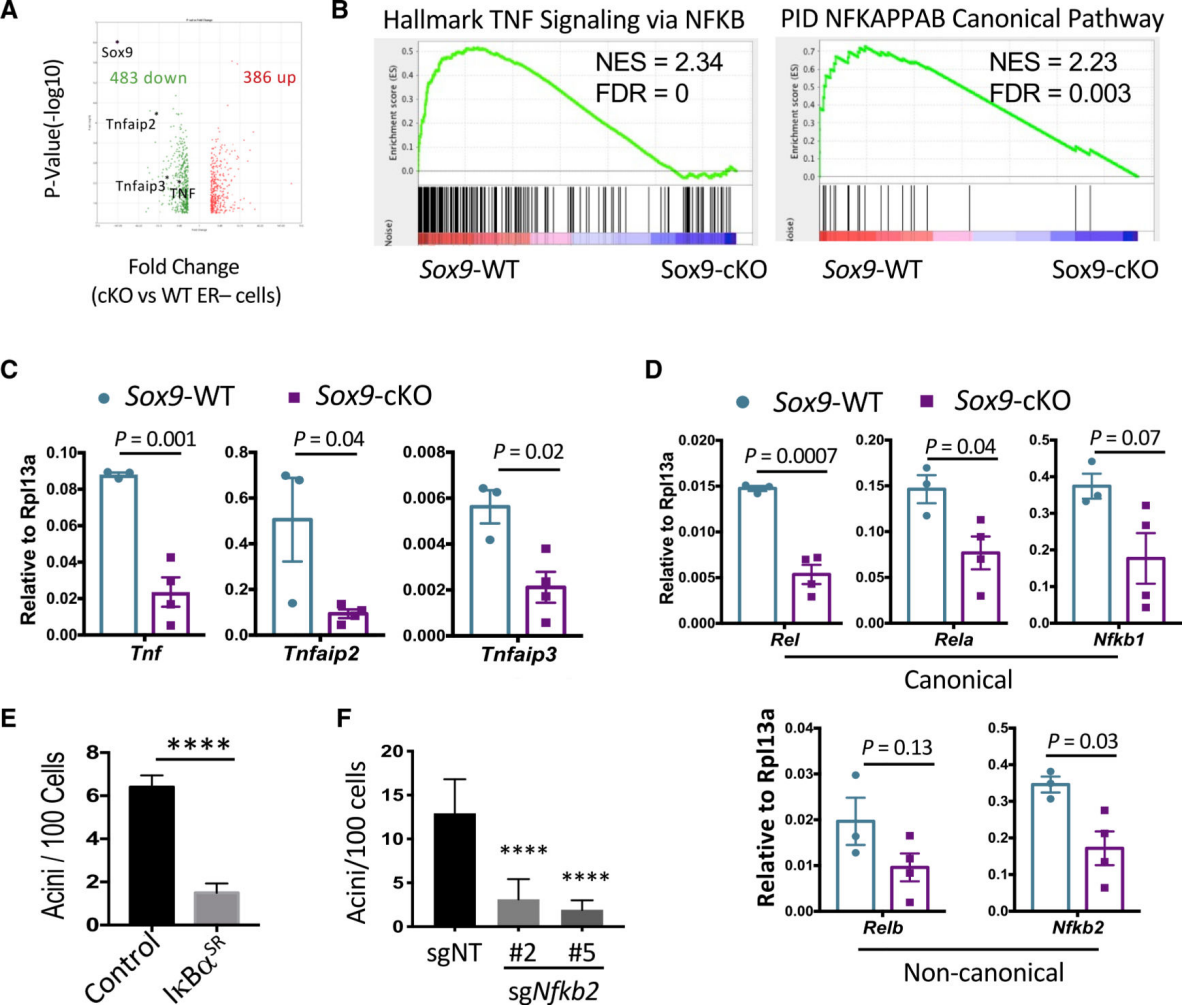
*Sox9* Deletion Leads to NF-κB Signaling Defect *In Vivo* (A) Volcano plot comparing the transcriptomes of *Sox9-*WT (*Sox9*^F/F^) and *Sox9-*cKO (MMTV-iCre; *Sox9*^F/F^) ER^−^ luminal cells. Genes with >2-fold expression difference and p < 0.05 were plotted. Total RNA of sorted ER^−^ luminal cells (n = 3 mice per group, 8–12 weeks of age in diestrus) was used for microarray analysis. (B) GSEA analysis showing enrichment of representative NF-κB-related gene sets in *Sox9-*WT ER^−^ luminal cells compared to *Sox9*-cKO cells. (C) Validation of NF-κB target genes in ER^−^ luminal cells sorted from either *Sox9*-WT or *Sox9*-cKO animals by qRT-PCR (n = 3–4 per group, 8–12 weeks of age in diestrus). Mean ± SEM. (D) Expression levels of NF-κB family transcription factors in *Sox9*-WT or *Sox9*-cKO ER^−^ luminal cells, as measured by qRT-PCR (n = 3–4 per group, 8–12 weeks of age in diestrus). Mean ± SEM. (E) Acinus-forming ability of ER^−^ luminal cells transduced with the IκBα^SR^ or control vector. FACS-sorted cells from C57BL/6J were transduced in organoid culture, puromycin selected, and then reseeded for measuring acinus-forming ability. Representative results of two experiments are shown. Mean ± SEM. ****p < 0.0001. (F) Acinus-forming ability of Rosa26-Cas9 ER^−^ luminal cells transduced with lentiviral vectors expressing *Nfkb2*-targeting or control non-targeting guide RNAs. FACS sorted cells were transduced in organoid culture, puromycin selected, and then reseeded for measuring acinus-forming ability. Two *Nfkb2*-targeting sgRNAs were used to control specificity. Representative results of two experiments are shown. Statistical analysis was performed by one-way ANOVA with Tukey multiple comparison correction. Mean ± SEM. ****p < 0.0001.

To understand downstream pathways mediating the function of SOX9, we compared the transcriptomes of *Sox9*-cKO and *Sox9*-WT ER^−^ luminal cells (Figure 2A). Gene set enrichment analysis (GSEA) of the transcriptomic data revealed that multiple nuclear factor κB (NF-κB) pathway related gene sets were among the most significantly downregulated molecular signatures in *Sox9*-cKO cells (Figures 2B and S2A). The downregulation of NF-κB signaling was further confirmed by qRT-PCR measuring NF-κB target genes, including *Tnf*, *Tnfaip2*, and *Tnfaip3* (Figure 2C). Interestingly, loss of *Sox9* significantly downregulated multiple members in the NF-κB family, including ones regulating the canonical (*Rel*, *Rela*, and *Nfkb1*) and non-canonical (*Relb* and *Nfkb2*) pathways (Figure 2D). We surmise that the downregulation of these NF-κB transcription factors likely contributes to the inhibition of NF-κB signaling in *Sox9*-cKO cells.

To determine the function of NF-κB signaling in ER^−^ LSPCs, we first transduced ER^−^ luminal cells with an IkBa superrepressor mutant (IκBα^SR^) that constitutively inhibits canonical NF-κB signaling (Van Antwerp and Verma, 1996). Expression of IκBα^SR^ markedly inhibited acinus formation by ER^−^ cells (Figure 2E). We then inhibited the non-canonical NF-κB pathway by CRISPR-mediated knockout of *Nfkb2*, thereby preventing the formation of the transcriptionally active NFKB2/RELB complex. Knockout of *Nfkb2* with two independent sgRNAs significantly inhibited the acinus-forming activity in ER^–^ luminal cells (Figure 2F). These results suggest that both canonical and non-canonical NF-κB signaling are required to maintain ER^−^ LSPC activity. Supporting this notion, GSEA analysis of our recently published single-cell RNA-seq data (Chung et al., 2019; Giraddi et al., 2018) demonstrated that NF-κB signaling was significantly upregulated in ER^−^ luminal cells (Figure S2B). In addition, canonical and non-canonical NF-κB pathways are both activated in ER^−^ LSPCs in *BRCA1* mutation driven tumorigenesis (Nolan et al., 2016; Sau et al., 2016). SOX9 likely potentiates the response of ER^−^ LSPCs to NF-κB stimuli by increasing the level of NF-κB family transcription factors.

### Human BLBC Expresses High Levels of *SOX9*

As previously mentioned, BLBC is likely to originate from the ER^−^ luminal lineage (Lim et al., 2009; Molyneux et al., 2010). Thus, we examined potential correlations between SOX9 and BLBC using gene expression datasets from The Cancer Genome Atlas (TCGA) BRCA and METABRIC studies (Ciriello et al., 2015; Koboldt et al., 2012; Pereira et al., 2016). In both datasets, *SOX9* expression is significantly upregulated in BLBC compared to other molecular subtypes (Figure S2C). Using the TCGA dataset, we found that higher *SOX9* levels in tumor samples correlated with greater enrichment of the molecular signatures of normal ER^−^ luminal cells and ER^−^ tumors (Figure S2D), in agreement with a previous study with a small number of tumor samples (Domenici et al., 2019). Further supporting the SOX9 association with ER^−^ tumors, we found that *SOX9*-high (*Z* score ≥ 2) breast cancer had significantly decreased ESR1 at both the RNA and protein levels (Figure S2E; Cerami et al., 2012; Gao et al., 2013). Additionally, high *SOX9* levels are associated with shorter relapse-free survival within BLBC patients in two large independent datasets (Figure S2F; Jé zé quel et al., 2012, 2013; Nagy et al., 2018). These findings suggest a potential functional role of SOX9 in BLBC pathogenesis.

### SOX9 Upregulation Is Required for Lineage Plasticity Caused by Loss of BLBC Tumor Suppressors

**Figure 3.**
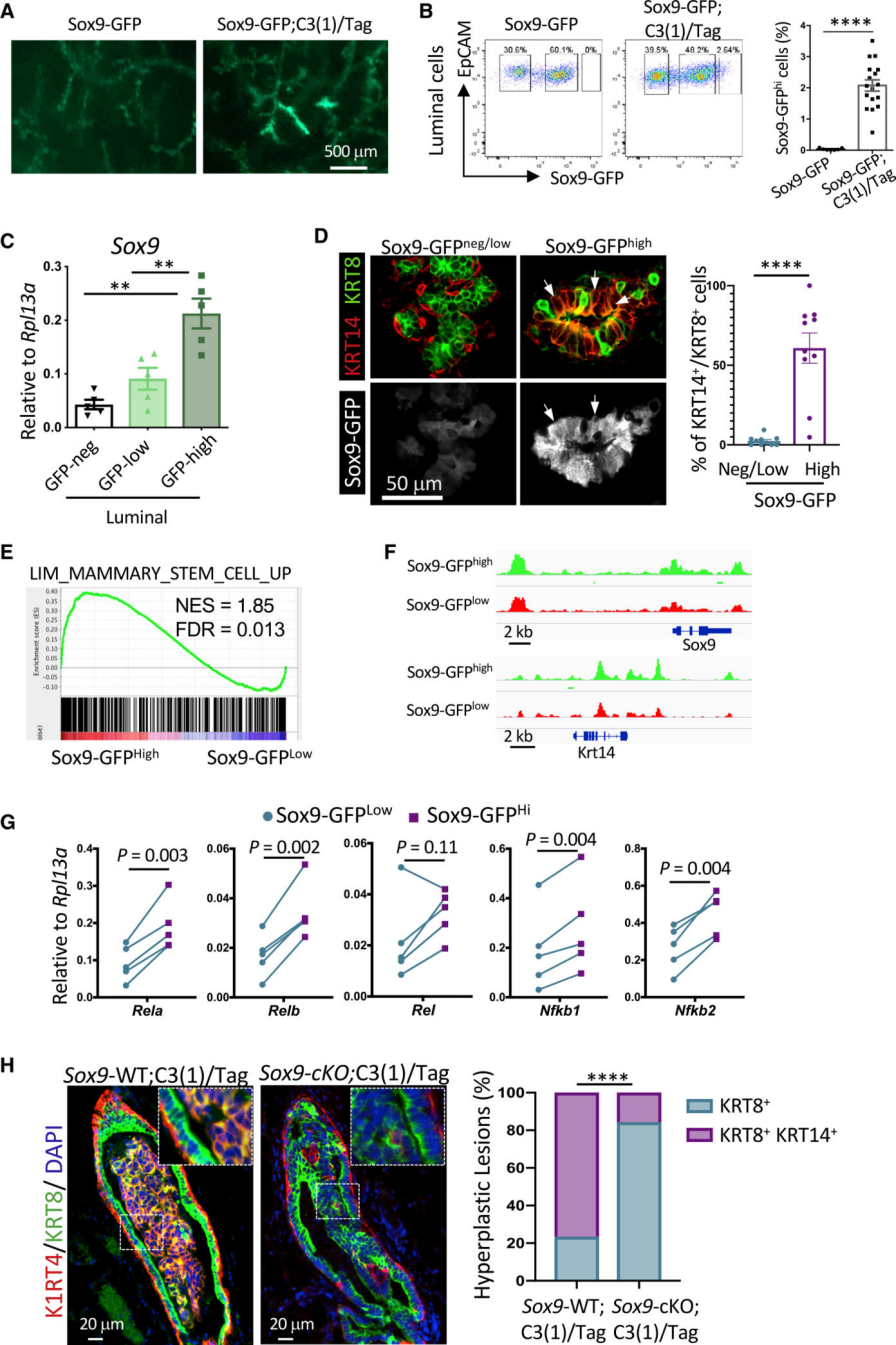
Upregulation of *SOX9* during C3(1)/Tag BLBC Tumorigenesis Results in Luminal-to-Basal Reprogramming (A) Representative GFP whole-mount images of inguinal mammary glands from either Sox9-GFP (n = 7) or Sox9-GFP;C3(1)/Tag (n = 17) animals at ~3 months of age. (B) Sox9-GFP levels in luminal cells from Sox9-GFP (n = 7) or Sox9-GFP;C3(1)/Tag (n = 17) mice at rv3 months old. Mean ± SEM. ****p < 0.0001. (C) qRT-PCR quantification of *Sox9* levels in luminal cells various levels of GFP. Cells were FACS-sorted from Sox9-GFP; C3(1)/Tag animals (n = 5). Mean ± SEM. **p < 0.01. (D) KRT8 (Alexa Fluor 546) and KRT14 (Alexa Fluor 647) immunostaining of mammary gland cryosections from ~3-month-old Sox9-GFP; C3(1)/Tag animals. Representative images of Sox9-GFP^neg/low^ or Sox9-GFP^high^ ducts are shown (left). The arrows point to representative KRT8^+^/KRT14^+^/Sox9-GFP^high^ cells. The graph shows the percentage of Sox9-GFP^neg/low^ or Sox9-GFP^high^ cells exhibiting the KRT8^+^/KRT14^+^ phenotype in each duct. Mean ± SEM. ****p < 0.0001. (E) GSEA comparing transcriptomes of Sox9-GFP^High^ and Sox9-GFP^Low^ luminal cells of rv3-month-old Sox9-GFP;C3(1)/Tag animals (n = 3). (F) ATAC-seq gene tracks for *Sox9* and *Krt14* in Sox9-GFP^low^ or Sox9-GFP^high^ luminal cells from rv3-month-old Sox9-GFP;C3(1)/Tag mice (n = 2 biological samples, four animals per sample). (G) qRT-PCR measuring the expression levels of NF-κB factors in Sox9-GFP^low^ and Sox9-GFP^high^ luminal cells FACS sorted from ~3-month-old Sox9-GFP;C3(1)/Tag mice (n = 5). (H) KRT8 (Alexa Fluor 568) and KRT14 (Alexa Fluor 488) immunostaining of hyperplastic lesions in either *Sox9*-WT;C3(1)/Tag (Sox9^F/F^; C3(1)/Tag) or *Sox9*-cKO;C3(1)/Tag (MMTV-iCre; *Sox9*^F/F^; C3(1)/Tag) animals at 6–7 months of age. Representative images (left) and quantification (right) of the lesion phenotypes are shown. 30 lesions from four *Sox9*-WT;C3(1)/Tag and 19 lesions from five *Sox9-*cKO;C3(1)/Tag mice were analyzed. Statistical analysis was performed by Fisher’s exact test. ****p < 0.0001.

To investigate the role of *Sox9* in BLBC, we crossed the C3(1)/Tag BLBC mouse model to a Sox9-GFP transgenic reporter mouse strain (Gong et al., 2010; Green et al., 2000; Maroulakou et al., 1994). The C3(1)/Tag model expresses SV40 large T antigen, which induces tumorigenesis by inactivating TP53 and RB (Ali and DeCaprio, 2001; Green et al., 2000), two of the most frequently mutated tumor suppressors in human BLBC. Interestingly, we found that Tag in the C3(1)/Tag model was primarily expressed in ER^−^ luminal cells, the likely origin of BLBC (Figure S3A). Furthermore, it faithfully recapitulates the multi-step progression and transcriptomic profiles of human BLBC (Pfefferle et al., 2013). As expected, Sox9-GFP is expressed in a fraction of luminal cells in the control mammary gland (Figures 3A and 3B). Interestingly, there was a pronounced increase of Sox9-GFP in focal regions of mammary ductal trees in C3(1)/ Tag animals starting at 2–3 months of age (Figure 3A). This increase was further demonstrated by the appearance of a Sox9-GFP^high^ population in luminal cells (Figure 3B). In contrast, the Sox9-GFP levels in C3(1)/Tag basal cells were not significantly changed (Figure S3B). We validated that Sox9-GFP^high^ cells expressed significantly elevated levels of SOX9 mRNA and protein (Figures 3C and S3C). This indicates that inactivation of TP53 and RB by SV40 large T antigen may directly or indirectly enable upregulation of *Sox9* in luminal cells *in vivo*.

As mentioned above, luminal cells acquire basal cell features during BLBC tumorigenesis (Lim et al., 2009; Molyneux et al., 2010; Wang et al., 2019). Therefore, we characterized the lineage status of Sox9-GFP^high^ cells based on the expression of luminal (KRT8) and basal (KRT14) keratins. Interestingly, prior to overt tumor formation, some areas of C3(1)/Tag mammary glands started to produce KRT8 and KRT14 double-positive (KRT8^+^/KRT14^+^) cells (Figure 3D). Similar bipotent cells have been observed in fetal mammary glands and during neoplastic transformation driven by *Pik3ca*^H1047R^ mutation and *Brca1* deletion (Giraddi et al., 2018; Van Keymeulen et al., 2015; Koren et al., 2015; Spike et al., 2012; Wang et al., 2019). Remarkably, these KRT8^+^/KRT14^+^ cells were almost entirely SOX9-GFP^high^, as shown by immunostaining on tissue sections and flow cytometry (Figures 3D and S3D). Additionally, transcriptomic analysis of Sox9-GFP^high^ and Sox9-GFP^low^ luminal cells in premalignant C3(1)/Tag mammary glands showed a significant enrichment of basal cell molecular signatures in Sox9-GFP^high^ cells, which was further validated by qRT-PCR (Figures 3E and S3E). Interestingly, the Sox9-GFP^high^ cells exhibited a higher proliferation rate than other luminal cells, supporting their role in tumor initiation (Figures S3E and S3F).

We further investigated whether Sox9-GFP^high^ cells underwent chromatin-landscape reprogramming toward the basal cell state by assay for transposase-accessible chromatin using sequencing (ATAC-seq) (Buenrostro et al., 2013). Compared to Sox9-GFP^low^ cells, Sox9-GFP^high^ cells had 1181 more-accessible chromatin regions and only 14 less-accessible regions (Figure S3G). As expected, the proximal promoter and distal regions around the *Sox9* locus was more accessible in Sox9-GFP^high^ cells (Figure 3F). Furthermore, reflecting the KRT14^+^ nature of Sox9-GFP^high^ cells, the chromatin regions around *Krt14* were also more accessible (Figure 3F). Concomitantly, the expression levels of both genes were increased in Sox9-GFP^high^ cells (Figures 3C and S3E). To further understand the genome-wide chromatin organization change, we compared Sox9-GFP^high^ and Sox9-GFP^low^ cells for the chromatin accessibility of uniquely accessible regions (UARs) that we recently described in basal cells, ER^−^ luminal cells, and ER^+^ luminal cells (Dravis et al., 2018). Interestingly, chromatin at the basal UARs became more accessible in Sox9-GFP^high^ cells, whereas luminal UARs were not significantly changed between Sox9-GFP^high^ and Sox9-GFP^low^ cells (Figure S3H). Analysis of the ATAC-seq data with chromVAR, a bioinformatic tool for inferring transcription factor (TF) activity using chromatin-accessibility data (Schep et al., 2017), suggested that Sox9-GFP^high^ cells had increased canonical and non-canonical NF-κB activity (Figure S3I). We also found that Sox9-GFP^high^ cells expressed higher levels of NF-κB TFs than Sox9-GFP^low^ cells (Figure 3G), consistent with the role of SOX9 in upregulating NF-κB TFs (Figure 2D).

To determine the functional role of SOX9 in luminal-to-basal reprogramming, we conditionally knocked out *Sox9* in the C3(1)/Tag model by crossing *Sox9*-cKO animals to the C3(1)/ Tag model. Analysis of hyperplastic lesions revealed that while most lesions in the control C3(1)/Tag mice contained KRT8^+^/KRT14^+^ cells, the great majority of lesions in *Sox9*-cKO C3(1)/ Tag mice remained as KRT8^+^ (Figure 3H). These results suggest that Sox9 upregulation enabled by TP53 and RB inactivation promotes luminal-to-basal reprogramming to generate a transitory hybrid cell state during BLBC tumorigenesis.

### *Sox9* Deletion Inhibits the Progression of Benign Lesions to Invasive Tumors in the C3(1)/Tag Model

**Figure 4.**
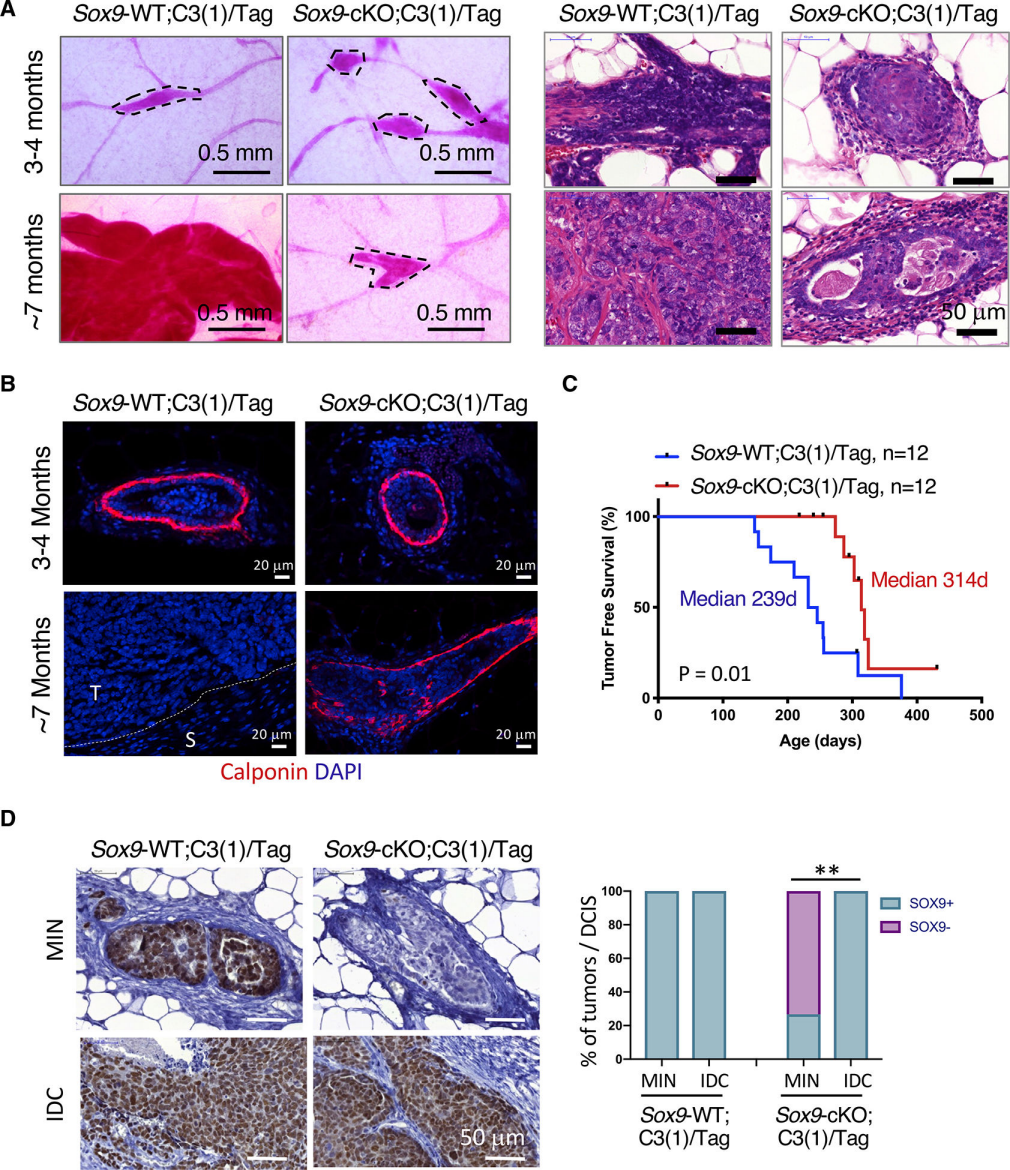
*Sox9* Deletion Inhibits the Progression of Benign Lesions to Invasive Carcinoma (A) Representative carmine whole-mount (left) and hematoxylin and eosin (right) staining of mammary glands or tumors from *Sox9*-WT;C3(1)/Tag or *Sox9*-cKO;C3(1)/Tag mice (n = 5 per group). (B) Representative calponin stain in MINs or tumors from the indicated animals (n = 3–5 per group). The dotted line marks the interface of tumor area (T) and stromal area (S). (C) Kaplan-Meier survival curve showing tumor onset in *Sox9*-WT;C3(1)/Tag or *Sox9*-cKO C3(1)/Tag animals (n = 12 per group). p value was calculated by log-rank test. (D) SOX9 immunohistochemistry (IHC) in MINs and invasive ductal carcinoma (IDC). 17 MINs from 4 *Sox9*-WT;C3(1)/Tag and 30 MINs from 7 *Sox9*-cKO;C3(1)/Tag mice were analyzed. p value was determined by Fisher’s exact test. **p < 0.01.

Utilizing the *Sox9*-cKO; C3(1)/Tag mice, we further investigated the effect of *Sox9* deletion on BLBC progression. When examined at 3–4 months of age, *Sox9*-cKO and control C3(1)/Tag mice showed no significant difference in the formation of mammary intraepithelial neoplasia (MIN), noninvasive lesions similar to ductal carcinoma *in situ* (DCIS) in human breast cancer, indicating *Sox9* deficiency does not affect early-stage hyperplasia (Figures 4A and 4B). The Tag expression levels were also similar between WT and *Sox9* null MINs (Figure S4A). At ~7 months of age, ~40% of the control C3(1)/Tag mice had developed palpable tumors (Figure 4C), which exhibited invasive histology and a disrupted myoepithelial layer as shown by calponin staining (Figures 4A and 4B). However, the MINs in ~7-month-old *Sox9*-cKO C3(1)/Tag mice were arrested at a stage similar to that found in younger animals, exhibiting a noninvasive histology and an intact myoepithelial layer (Figures 4A and 4B).

Consequently, the *Sox9*-cKO; C3(1)/Tag animals had a significant delay in palpable tumor formation (Figure 4C). Although they did eventually develop palpable tumors, all tumors in *Sox9-*cKO;C3(1)/Tag mice still expressed high levels of SOX9 similar to the control C3(1)/Tag tumors (Figure 4D). Interestingly, in line with the incomplete penetrance of MMTV-iCre expression, ~25% of MINs in *Sox9*-cKO;C3(1)/Tag mice expressed SOX9 (Figure 4D). This suggests that these SOX9 WT “escapee” cells were responsible for invasive tumor formation in *Sox9*-cKO mice. Furthermore, deletion of the floxed *Sox9* allele in primary tumor cells from *Sox9-*WT;C3(1)/Tag or *Sox9-*cKO;C3(1)/Tag mice with an adenoviral Cre vector inhibited their tumor organoid-forming ability (Figure S4B). Taken together, the data support an essential role for Sox9 in the development of invasive C3(1)/Tag tumors.

## DISCUSSION

Understanding why LSPCs are prone to BLBC transformation and how they undergo lineage reprogramming will help develop preventive and therapeutic strategies for this aggressive cancer type. Our work reveals that the SOX9 transcription factor acts as a determinant of the ER^−^ LSPC fate and its upregulation enabled by loss of BLBC tumor suppressors contributes to lineage plasticity and the progression of benign lesions to invasive tumors during BLBC oncogenesis. These results demonstrate that SOX9, as an ER^−^ LSPC determinant, acts as a critical lineage plasticity driver for BLBC.

Previous studies have shown specific expression of SOX9 in the ER^−^ luminal lineage (Domenici et al., 2019; Wang et al., 2017). Using *in vivo* genetic approaches, we demonstrate that SOX9 is functionally required for the ER^−^ LSPC activity. Although *Sox9* null ER^−^ luminal cells can be maintained *in vivo*, they are devoid of LSPC activity as measured by *exvivo* organoid culture. The loss in LSPC activity is also manifested by a defect in alveologenesis during early pregnancy, although it was recovered by late pregnancy. We suggest that this is likely due to incomplete deletion of the floxed *Sox9* allele by MMTV-iCre in the mammary epithelium. Interestingly, similar compensation has been observed in a RANK conditional knockout study, in which complete RANK deletion by K5-Cre inhibited alveologenesis, but incomplete deletion by MMTV-Cre had no effect (Schramek et al., 2010).

We found that SOX9 promotes the activation of canonical and non-canonical NF-κB pathways and that both pathways are required for ER^−^ LSPC activity. Previous work has shown that NF-κB is activated in mammary ER^−^ luminal cells and that the NF-κB activators IKKα and RANK are required for alveologenesis (Cao et al., 2001; Fata et al., 2000; Pratt et al., 2009). Furthermore, both canonical and non-canonical NF-κB signaling is required for BLBC oncogenesis driven by BRCA1 mutation (Nolan et al., 2016; Sau et al., 2016; Sigl et al., 2016). SOX9 contributes to NF-κB signaling by increasing the expression of NF-κB family transcription factors (TFs) in ER^−^ LSPCs. This, together with elevated RANK expression in ER^−^ LSPCs (Nolan et al., 2016), is likely to make them hyper-responsive to RANK ligand and other NF-κB stimuli, therefore making these cells more susceptible to transformation.

Our work identified a key driver of lineage plasticity in BLBC. Inactivation of the BLBC tumor suppressors TP53 and RB in the C3(1)/Tag model (Ali and DeCaprio, 2001; Green et al., 2000) leads to a luminal-to-basal transition at the early stage of hyperplasia. Similar early lineage reprogramming has been observed in *Pik3ca*- and *Brca1*-mutation-driven mammary tumorigenesis (Van Keymeulen et al., 2015; Koren et al., 2015; Wang et al., 2019). These findings suggest lineage reprogramming is likely a common mechanism for generating transformation-competent transitory cells in breast oncogenesis. Interestingly, TP53 and RB inactivation by the SV40 Tag enables the upregulation of SOX9 in ER^−^ LSPCs, and SOX9 is required for lineage reprogramming. It is worth noting that although the SV40 Tag was expressed throughout the mammary ducts, SOX9-high cells only emerge at focal regions. This suggests that Tag is like to cooperate with other cell-intrinsic or cell-extrinsic signals to upregulate SOX9. SOX9 has been shown to function as a pioneer factor that can reshape the chromatin landscape (Adam et al., 2015). Consistent with this, we found that SOX9-high luminal cells had an increased number of open chromatin regions that were enriched in areas we previously defined as UARs in basal cells (Dravis et al., 2018). Thus, SOX9-high cells exhibit chromatin-landscape features of both luminal and basal cells. It is worth noting that our work also uncovered a useful tool for studying the transitory reprogramming of cells that can be isolated using the Sox9-GFP reporter, adding to other recently developed reporters for potential basal and luminal bipotent cells (Dravis et al., 2018; Sonzogni et al., 2018).

We found that SOX9 has a particularly important role in the progression of benign lesions to invasive BLBC tumors. In the C3(1)/ Tag model, *Sox9* deletion did not impair the formation of MINs, lesions similar to DCIS in human. However, the *Sox9* null MINs failed to progress to invasive tumors, and the only tumors formed in the *Sox9*-cKO;C3(1)/Tag mice were from cells that escaped *Sox9* deletion. These results revealed a role for SOX9-mediated perturbations of the stem/progenitor cell program in DCIS progression. One possible explanation for this phenotype is that SOX9 acts in cooperation with another SOXE factor, SOX10, in this process based on the following observations. We have recently reported that SOX-binding motifs, including those of SOX10 and SOX9, are enriched in chromatin that is uniquely accessible in stem/progenitor cells (Dravis et al., 2018). SOX10 overexpression also induces reprogramming of cells in multiple mammary cancer models to an invasive, mesenchymal-like state that closely resembles a neural crest-like state (Dravis et al., 2018). Thus, Sox10 and Sox9 overexpression exhibit similar invasive phenotypes, which we speculate contributes to DCIS progression. Interestingly, we found that SOX9 ectopic expression can induce SOX10 expression, and studies have shown that SOXE factors can function as heterodimers (Huang et al., 2015). This suggests that SOX9 and SOX10 are likely to act cooperatively to regulate cell fate plasticity in mammary stem/progenitor cells and breast cancer progression. Future studies are needed to determine whether these factors are correlated with faster DCIS progression in human patients.

## STAR★METHODS

### RESOURCE AVAILABILITY

#### Lead Contact

Further information and requests for resources and reagents should be directed to and will be fulfilled by the lead contact, Dr. Wenjun Guo (wenjun.guo@einsteinmed.org).

#### Materials Availability

Materials produced in this study are available upon request and with a completed MTA.

#### Data and Code Availability

The accession number for the microarray and ATAC-seq data reported in this paper is GEO: GSE135892.

### EXPERIMENTAL MODEL AND SUBJECT DETAILS

#### Mice

Sox9^Flox^ (JAX # 013106) (Akiyama et al., 2002), C3(1)/Tag (JAX # 013591) (Green et al., 2000; Maroulakou et al., 1994) and Rosa26-Cas9 (JAX # 026179) (Platt et al., 2014) were obtained from the Jackson Laboratory. MMTV-iCre (MMTV-iCre/CAG-CAC-ECFP) mice were provided by Dr. Jeffrey Pollard (Roussos et al., 2010). Mice for Sox9 conditional knockout experiments were generated by crossing Sox9^Flox/Flox^; MMTV-iCre with Sox9^Flox/Flox^; C3(1)/Tag or Sox9^Flox/+^; MMTV-iCre with Sox9^Flox/+^ mice on a C57BL/6J x FVB/NJ mixed background. Sox9-GFP transgenic mice (Tg(Sox9-EGFP)EB209Gsat/Mmucd) were obtained from Mutant Mouse Resource & Research Centers (MMRRC) and backcrossed to FVB/NJ, which were then crossed with C3(1)/Tag in FVB/NJ.

Genotyping was performed using the primers listed in Table S1. Tg(Sox9-EGFP)EB209Gsat mice were phenotyped by EGFP expression in ear punch hair follicles under a fluorescent microscope.

All experimental animals were females at 2–4 months of age or as indicated in the Results, Figures and Figure Legends.

All experimental procedures were performed in accordance with protocols approved by the Institutional Animal Care and Use Committee of Albert Einstein College of Medicine. All experimental animals were female and SPF as determined by testing of sentinel animals.

### METHOD DETAILS

#### Mammary Epithelial Cell Single Cell Preparation

Number three and number four mammary fat pads were dissected and minced until no piece was larger than the bore of a 10 mL serological pipet. For every complete set of glands, 3 mL of the DMEM/F12 (Corning 10–092-CV) + 300 units/ml Collagenase 3 (Worthington LS004182) + 10 units/ml DNase I (Worthington LS002139) + 5 μM Y-27632 (Caymen Chemical 10005583) was used. This mixture was incubated for 2 hours rocking at 37°C. After primary digestion the epithelial pellet was washed with 1x PBS, RBCs were lysed, and RBC lysis buffer was removed. 1 mL of 0.05% Trypsin-EDTA (ThermoFisher 25300054) was then added (for up to three animals), mixed, and incubated for 5 minutes at 37°C. Trypsinization was halted with serum containing media, the sample was triturated, and the cells were pelleted again. This final pellet was incubated 1 mL of DMEM/F12 with 1 U/ml Dispase (Worthington LS02109) with 100 U/ml DNase (Worthington LS002139) at 37°C for five minutes. Dispase was halted by dilution and the final suspension was passed through a 40-micron filter.

#### Fluorescence Activated Cell Sorting (FACS) and Analysis

Fluorescently conjugated antibodies were used 1:100 for all experiments. Staining was accomplished by incubating fluorescently conjugated antibodies for 30 minutes inside a 4°C refrigerator. Keratin 14 was stained for flow cytometry by first fixing cells already stained for surface markers with 3.2% paraformaldehyde (EMS 15714) in PBS for 15 minutes. Fixed cells were then permeabilized with 0.1% Triton X-100 for 10 minutes. Cells were then spun out of permeabilization buffer and resuspended in FACS staining buffer where they were stained for 15 minutes with unlabeled rabbit anti-Keratin 14 (1:1000) followed by 15 minutes with anti-rabbit Alexa Fluor 647 (1:100).

All sorting was performed on a BD Bioscience FACSAriaII, and all analysis was performed on BD LSRII. Analysis of FCS files was done using FlowJo version 10.4.2.

#### Organoid Culture

FACS sorted mammary epithelial cells was resuspended in ice cold Epicult-B media + 5% FBS + 10 ng/ml EGF + 20 ng/ml FGF2 + 4 mg/ml Heparin + 5 μM Y-27632 + 5% Matrigel (Corning 354234). This mixture was then plated on 96-well ultra-low attachment plate (Corning 3474) (10–1,000 cells /well), or on 2-hydroxyethyl methacrylate (Poly-HEMA) coated 6- and 24-well plates (10,000–200,000 cells/well). For organoid passaging, organoids were collected, washed with PBS, disassociated with 0.05% Trypsin-EDTA and then reseeded in organoid culture medium.

#### Lentivirus Production and Infection

Lentivirus was produced in 293T cells using pMD2.G capsid plasmid and pCMVR8.74 packaging plasmid, a gift from Didier Trono (Addgene Plasmids #12259 and 22036). Lentivirus was either used straight or concentrated 10–100x using Lenti-X Concentrator (Takara 631232) as described in the manufacturer’s instructions. Lentiviral infection of ER^−^ luminal cells in suspension was carried out by adding virus (no greater than 50% of total media volume) to single cells in organoid medium with the addition of 5 μg/ml of Polybrene (EMD Millipore TR-1003-G) and culturing overnight. The next morning the cells were washed to remove Polybrene and lentivirus. The washed cells were then reseeded back into organoid medium.

#### Mammary Gland Whole Mount and Carmine Staining

The number four mammary gland was dissected out and stretched across a Superfrost glass slide (Fisher Scientific 12–550-15). The gland was then fixed in Carnoy’s solution. The gland was placed in carmine-alum solution and allowed to stain overnight. The following day, the slide mounted gland was dehydrated and destained. The slide mounted gland was then immersed in xylenes (Fisher Scientific X3P-1GAL), allowed to become transparent, and mounted with a generous portion of Permount (Fisher Scientific SP15100).

#### Estrus Cycle Determination

Mice were restrained with one hand and in the other a 0.5–10 μL pipette was preloaded with 10 μL of sterile, room temperature, 1x PBS. The vagina of the animal was then flushed with the loaded 10 μL of 1x PBS after which that same 10 μL was recovered with the pipette and added to a labeled tube. Each sample was then streaked on a slide and the cytology of the vaginal smear was evaluated using a phase contrast microscope at 10x magnification. Estrus cycle was determined based on cytology (Caligioni, 2009).

#### Total RNA Isolation from Flow Sorted Cells

Cells were sorted directly into 0.75 mL of Trizol LS (ThermoFisher 10296010) as processed as per the Trizol LS protocol. RNA was quantified, as per manufacturer’s instructions, with Qubit® RNA HS Assay Kit (ThermoFisher Q32852).

#### Quantitative RT-PCR

cDNA was synthesized from either poly-A selected or total RNA as per manufacturer’s instructions with the High-Capacity cDNA Reverse Transcription Kit (ThermoFisher 4368813).

RT-PCR reactions were performed using Power SYBR® Green PCR Master Mix (ThermoFisher 4368708). Primers are listed in Table S2.

#### Microarray Analysis of Sox9^Fl/Fl^ and Sox9^Del/Del^ ESR1^−^ Luminal Cells

Estrus cycle of Sox9^Fl/Fl^ and MMTV-iCre; Sox9^Fl/Fl^, between 8–12 weeks, was tracked Gene expression profiling was done using total RNA on an Affymetrix Clariom S Pico, Mouse (ThermoFisher 902932) by the Einstein Genomics Core Facility as per manufacturer’s instructions. CHP files were converted to CEL files using Affymetrix Expression Console. The resulting CEL files were then analyzed using the Transcriptome Analysis Console (TAC) version 4.0.1. The expression data was then output as a human readable text file and reformatted as an GCT file manually.

#### Gene Set Enrichment Analysis of ESR1- Luminal Cell Microarray Data

The GCT file, generated from the microarray data, was loaded into Gene Set Enrichment Analysis (GSEA) version 3.0 (Mootha et al., 2003; Subramanian et al., 2005). Phenotype labels were done with Sox9^Fl/Fl^ samples being set as “Class A” while Sox9^Del/Del^ were set as “Class B.” GSEA analysis was run using the default settings except for “Permutation type” which was set to “gene set.” The gene sets V6.1 H1 Hallmarks and C2 CGP and CP were used for analysis. A false discovery rate of 0.25 was used as cutoff for significance.

#### Gene Set Enrichment Analysis of TCGA Breast Cancer RNA-Seq Data

Raw HTseq count files from all samples in the BRCA database were downloaded from the NIH GDC Data portal. These files were merged into a single GCT file using MergeHTSeqCounts version 0.7 on the Broad GenePattern server (Reich et al., 2006). The GCT file was then preprocessed using PreprocessReadCounts version 0.6 on the Broad GenePattern server. Preprocessing removes lowly expressed genes and then normalizes the dataset to make it better resemble microarray data for which GSEA was designed. This preprocessed GCT file was then loaded into GSEA. The gene sets V6.1 H1 Hallmarks and C2 CGP and CP were used for analysis. Sox9 was used as a gene phenotype and the metric for ranking genes was set to Pearson. A false discovery rate of 0.25 was used as cutoff for significance.

#### Single-cell RNA-Seq Analysis and Gene Set Enrichment Analysis

Fastq files from scRNA-seq were aligned and quantified with the Alevin function of Salmon v0.13. Gene-Cell matrices were turned into a Seurat object, normalized with SCTransform analyzed by Seurat v3.0 (Hafemeister and Satija, 2019; Stuart et al., 2019). Differentially expressed genes between ER- luminal and all other clusters were determined by the Wilcoxon test within Seurat with a cutoff of FDR ≤ 0.05 and then exported and ranked based on their log fold change. This rank file was then used for preranked gene set enrichment analysis.

#### CRISPR sgRNA Design and Cloning

Sox9 and Nfkb2 sgRNAs were designed using the CHOPCHOP webserver (Labun et al., 2016). Oligos were cloned into pXLV-Puro as described previously (Cong et al., 2013; Zhang et al., 2016). The sgRNA targeting sequences are in Table S3.

#### Western Blots

All cells were lysed in RIPA buffer with protease and phosphatase inhibitors and sonicated in a Bioruptor (Diagenode). Protein samples were reduced with TCEP and separated on 4%–12% gradient TruPAGE Precast Gels (Sigma PCG2003) buffered by TruPAGE Tris-MOPS SDS Express Running Buffer (Sigma PCG3003) and wet tank transferred to a PVDF membrane (EMD Millipore IPVH00010). Membrane was blocked with 5% non-fat dry milk in TBS-T. Primary antibodies were incubated with the membrane overnight in 5% BSA TBS-T at indicated ratios. Secondary antibodies were incubated in 5% milk with TBS-T for one hour at RT. Between all antibody steps membrane was washed 3x for 5 minutes with TBS-T. The membrane was then incubated with either Western Lightning ECL Pro (PerkinElmer NEL121001EA) or VisiGlo Prime HRP Chemiluminescent ECL Substrate (Amresco 89424–016) depending on the sensitivity required. Membrane was then imaged via CCD camera using an Odyssey® Fc (Li-Cor). Resulting exposures were exported at 600 DPI in TIF format.

#### Tissue Fixation for Paraffin Embedding

The number 4 mammary fat pad of the mouse was dissected out and stretched across a Superfrost glass slide (Fisher Scientific 12–550-15). Tumors were dissected out and cut with a razor blade such that at least one side was at most 2 mm thick. These tissues were then place in 10% formalin and allowed to fix overnight at room temperature after which they were transferred to 70% ethanol. All paraffin embedding, and sectioning was performed by Einstein Histology & Comparative Pathology Core.

#### Processing and Staining of Formalin Fixed Paraffin Embedded Slides (FFPE)

Slides were rehydrated serially with a xylene:ethanol:water series. If a peroxidase reaction will be used as part of the staining pro- tocol, endogenous peroxidase was quenched with methanol (EMD Millipore MX04881) containing 0.3% hydrogen peroxide (Fisher Scientific NC0512932) for 15 minutes after the 100% ethanol step. Antigen retrieval was performed in a pressure cooker using 1x Antigen Unmasking Solution (Vector Laboratories H-3300). Each section was then blocked with TBS-T(0.1%) + 5% goat serum. Primary antibody was diluted in blocking solution and allowed to stain overnight at 4°C. After primary incubation the slide was washed 3x with TBS-T(0.1%) and stained either with a secondary that was conjugated with HRP or a secondary conjugated to a fluorophore. HRP signal was developed using DAB Peroxidase (HRP) Substrate Kit, 3,3′-diaminobenzidine (Vector Laboratories SK-4100) for a measured amount of time as required to see sufficient signal on a positive control slide. For fluorescently conjugated secondary antibodies the slide was incubated with secondary antibodies, diluted in 5% goat serum in TBS-T.

#### Imaging of FFPE Slides

Fluorescent slides were imaged with an AXIO Examiner D1 microscope (Zeiss), equipped with a confocal scanner unit CSU-X1 (Yakagawa), and automated micrometer stage (Sutter Instrument). Slidebook software version 6 was used for acquisition and flat field and dark field corrections were performed at the time of capture for all images.

Chromogen slides were scanned with a P250 High Capacity Slide Scanner (3D Histech).

#### Cloning of IκBα^SR^ into pLVX-Puro

IκBα^SR^ was PCR cloned from pBabe puro IkB alpha M, a gift from Inder Verma (Addgene plasmid # 12332), into pLVX-Puro (Takara 632164) via NEB HiFi Assembly Kit (New England Biolabs E2621S). PCR was performed using PrimeSTAR MAX DNA polymerase (Takara R045A) as per manufacturer’s instructions. Assembly reaction was transformed into ElectroMAX Stbl4 Competent Cells (ThermoFisher 11635018) using a MicroPulser Electroporator (BIO-RAD) using cuvettes with a 1 mm gap and the default settings for *E. coli*.

#### ATAC-seq and Data Analysis

The transposition assay was performed as previously described (Buenrostro et al., 2015) 1.4×10^4^ nuclei from sorted Sox9-GFP-high and Sox9-GFP-low C3(1)/Tag cells were used in each reaction with 20 mL of transposition mix (10 μL 2x TD, 0.5 mL TDE1, 6.6 ul PBS, 2.9 μL H2O; Illumina Nextera FC-121–1030) and incubated at 37°C for 30 minutes with shaking. The library was amplified with 12 cycles of PCR as determined by qPCR to be the optimal cycle number (25% library saturation). The library was purified with AMPure XP beads (Beckman A63881), and then analyzed by Agilent TapeStation, and 50 bp single-end sequencing was performed with Illu- mina HiSeq 2500. ATAC-seq analysis was performed as previously described (Bao et al., 2015). In brief, after quality check with FastQC, sequencing reads were mapped to the mouse genome (mm9) with Bowtie (Langmead et al., 2009), with these parameters: -m 1 -S -n 2 -l 30. Bedgraph files generated were then quantile normalized using the R package “preprocessCore” and converted into BigWig format and visualized in Integrative Genomics Viewer (Robinson et al., 2011). Genome-wide average signal profile at genes was checked for each sample to ensure similar signal-to-noise level. Signal profiling was performed using deepTools (Ramıŕez et al., 2014) Differential peaks were called with SICER-df-rb (Zang et al., 2009), with these parameters: window size: 100, gap size: 100, E-value: 0.01, FDR: 0.05.

### QUANTIFICATION AND STATISTICAL ANALYSIS

All statistics were performed as unpaired Student’s t test unless otherwise stated in the figure legend and using Prism version 7.0d. All details regarding n number and what n represents are stated in figure legends. A p value less than 0.05 was considered significant.

## Supplementary Material

1

## Figures and Tables

**KEY RESOURCES TABLE:** 

REAGENT or RESOURCE	SOURCE	IDENTIFIER
Antibodies		
Biotin anti-mouse TER-119	BioLegend	AB_313705
Biotin anti-mouse CD45	BioLegend	AB_312969
Biotin anti-mouse CD31	BioLegend	AB_312899
PerCP/Cy5.5 anti-mouse/human CD49f	BD Biosciences	AB_11151910
APC anti-mouse CD326 (Ep-CAM)	BioLegend	AB_1134102
PE/Cy7 anti-mouse Ly-6A/E (Sca-1)	BioLegend	AB_493596
PE Streptavidin	BioLegend	AB_2819020
Streptavidin V450	BD Biosciences	AB_2033992
Sox9 (D8G8H) Rabbit mAb	Cell Signaling Technology	AB_2665492
Rabbit Anti-Histone H3	Abcam	AB_302613
HRP Goat anti-mouse IgG	BioLegend 405306	AB_315009
HRP Goat anti-mouse IgG	BioLegend 406401	AB_2099368
Rabbit Purified anti-Keratin 14 Antibody	Biolegend 905304	AB_2616896
Chicken Purified anti-Keratin 14 Antibody	Biolegend 906004	AB_2616962
Rat Anti-Keratin 8 (Provided as Supernatant)	Developmental Studies Hybridoma Bank	AB_531826
Calponin 1 (D8L2T) XP	Cell Signaling Technology	AB_2798789
Goat anti-Rat IgG (H+L) Secondary Antibody, Alexa Fluor® 568 conjugate	ThermoFisher	AB_2534121
Alexa Fluor® 488-AffiniPure Goat Anti-Chicken IgY (H+L)	Jackson ImmunoResearch	AB_2337390
Alexa Fluor® 488-AffiniPure Goat Anti-Rabbit IgG (H+L)	Jackson ImmunoResearch	AB_2338046
Goat anti-Rabbit IgG (H+L) Cross-Adsorbed Secondary Antibody, Alexa Fluor 647	ThermoFisher	AB_2535812
Goat anti-Rat IgG (H+L) Cross-Adsorbed Secondary Antibody, Alexa Fluor 546	ThermoFisher	AB_2534125
Bacterial and Virus Strains		
ElectroMAX Stbl4 Competent Cells	ThermoFisher	Cat# 11635018
Chemicals, Peptides, and Recombinant Proteins		
Y-27632	Cayman Chemical	10005583
Phosphate-Buffered Saline, 1X without calcium and magnesium, pH 7.4 ± 0.1	Corning	21-040-CM
UltraPureTM 0.5M EDTA, pH 8.0	ThermoFisher	15575020
Human Recombinant Epidermal Growth Factor	Sigma	E9644
Human Recombinant Fibroblast Growth Factor 2	EMD Millipore	GF003
Ethanol	Decon Laboratories, Inc.	2701
Chloroform	Fisher Scientific	BP1145
Glacial Acetic Acid	Fisher Scientific	A38S500
1M Tris-HCl, pH 8.0	TekNova	T1080
Magnesium Chloride Hexahydrate	Fisher Scientific	BP214-500
Calcium Chloride	Fisher Scientific	BP510-500
EGTA	Sigma	E3889
Nonidet P-40 Substitute	IBI Scientific	IB01140
Sodium Deoxycholate	Sigma	30970
Sodium Fluoride	Alfa Aesar	11561-30
b-glycerophosphate disodium salt hydrate	Sigma	G9422
Sodium Orthovanadate	Sigma	S6508
Halt™ Protease Inhibitor Cocktails	ThermoFisher	PI87786
Red Blood Cell Lysing Buffer Hybri-Max	Sigma	R7757
Heparin	Sigma	H3149
Collagenase 3	Worthington	LS004182
DNase I	Worthington	LS002139
0.05% Trypsin-EDTA	ThermoFisher	25300054
Dispase	Worthington	LS02109
3.2% paraformaldehyde	EMS	15714
Triton X-100	Fisher Scientific	BP151
Matrigel	Corning	354234
Polybrene	EMD-Millipore	TR-1003-G
Xylenes	Fisher Scientific	X3P-1GAL
Permount	Fisher Scientific	SP15100
Trizol LS	ThermoFisher	10296010
TCEP	GoldBio	TCEP1
TruPAGE Tris-MOPS SDS Express Running Buffer	Sigma	PCG2003
Western Lightning ECL Pro	PerkinElmer	NEL121001EA
VisiGlo Prime HRP Chemiluminescent ECL Substrate	Amresco	89424-016
Methanol	EMD Millipore	MX04881
30% Hydrogen Peroxide	Fisher Scientific	NC0512932
Antigen Unmasking Solution	Vector Laboratories	H-3300
Lenti-X Concentrator	Takara	631232
DAB Peroxidase (HRP) Substrate Kit, 3,3′-diaminobenzidine	Vector Laboratories	SK-4100
Critical Commercial Assays		
High-Capacity cDNA Reverse Transcription Kit	ThermoFisher	4368813
Power SYBR® Green PCR Master Mix	ThermoFisher	4368708
PrimeSTAR MAX DNA polymerase	Takara	R045A
NEB HiFi Assembly Kit	New England Biolabs	E2621S
Qubit® RNA HS Assay Kit	ThermoFisher	Q3285
Affymetrix Clariom S Pico, Mouse	ThermoFisher	902932
TruPAGE Precast Gels	Sigma	PCG3003
Nextera DNA Library Kit	Illumina	FC-121-1030
Deposited Data		
Sox9-GFP; C3(1)-Tag Microarray Data, Sox9-cKO Microarray Data, and Sox9-GFP; C3(1)-Tag ATAC-seq Data	GEO	GEO: GSE135892
Experimental Models: Cell Lines		
Sox9-Flox Mammary Epithelial Organoids	This Paper	N/A
Rosa26-Cas9-EGFP Mammary Epithelial Organoids	This Paper	N/A
HEK293T cell line, ATCC	ATCC	CVCL_0063
Experimental Models: Organisms/Strains		
B6.129S7-Sox9^tm2Crm^/J *Mus musculus*	JAX	IMSR_JAX:013106
FVB-Tg(C3-1-TAg)cJeg/JegJ *Mus musculus*	JAX	IMSR_JAX:013591
B6J.129(Cg)-Gt(ROSA)26Sor^tm1.1(CAG-cas9_*_,-EGFP)Fezh^/J *Mus musculus*	JAX	IMSR_JAX:026179
FVB-Tg(MMTV-iCre/CAG-CAC-ECFP) *Mus musculus*	Pollard Lab	Roussos et al., 2010
STOCK Tg(Sox9-EGFP)EB209Gsat/Mmucd *Mus musculus*	MMRRC	MMRRC_011019-UCD
FVBJ.B6(Cg) Tg(Sox9-EGFP)EB209Gsat/Mmucd *Mus musculus*	This Paper	N/A
FVB-Tg(MMTV-iCre/CAG-CAC-ECFP); B6.129S7-Sox9^tm2Crm^/J *Mus musculus*	This Paper	N/A
FVB-Tg(MMTV-iCre/CAG-CAC-ECFP); B6.129S7-Sox9^tm2Crm^/J; FVB-Tg(C3-1-TAg)cJeg/JegJ *Mus musculus*	This Paper	N/A
FVB-Tg(C3-1-TAg)cJeg/JegJ; FVBJ.B6(Cg) Tg(Sox9-EGFP)EB209Gsat/Mmucd *Mus musculus*	This Paper	N/A
Oligonucleotides		
Primers for genotyping are found in the genotyping primers table	Multiple Sources	N/A
Primers for RT-PCR are found in the RT-PCR primers table	This Paper	N/A
Primers for cloning are found in the cloning primers table	This Paper	N/A
Recombinant DNA		
pBabe puro IkB alpha M	Addgene	Addgene_12332
pLVX-Puro	Takara	632164
pMD2.G	Addgene	Addgene_12259
pCMVR8.74	Addgene	Addgene_22036
pLVX-Puro-IKBKB^Mut^	This Paper	N/A
pXLV-Puro	Zhang et al., 2016	N/A
pXLV-sgNT1	This Paper	N/A
pXLV-Puro sgSox9	This Paper	N/A
pXLV-Puro sgNfkb2 E2	This Paper	N/A
pXLV-Puro sgNfkb2 E5	This Paper	N/A
Software and Algorithms		
TAC V4.0	Thermofisher	N/A
FlowJo VX	BD	N/A
GSEA 3.0	Mootha et al., 2003; Subramanian et al., 2005	N/A
Seurat V3.0	Stuart et al., 2019	N/A
Bowtie v1.2	Langmead et al., 2009	N/A
preprocessCore v1.46	GenePattern Server	N/A
deepTools v2.0	Ramírez et al., 2014	N/A
SICER-df-rb v1.1	Zang et al., 2009	N/A
BD FACSDiva V8	BD	N/A
chromVAR	Schep et al., 2017	N/A
Other		
EpiCult™-B Mouse Medium Kit	Stem Cell Technologies	05610
Fetal Bovine Serum, Heat Inactivated	Corning	35-015-CV
DMEM/F12	Corning	10-092-CV
AMPure XP beads	Beckman	A63881

## Highlights

- SOX9 is required for luminal stem/progenitor cell (LSPC) activity
- SOX9 enhances activation of canonical and non-canonical NF-κB pathways in LSPC
- SOX9 upregulation drives luminal-to-basal reprogramming during BLBC formation
- SOX9 deletion blocks progression of DCIS to invasive BLBC
