# Supplementary material for: Stem Cell Determinant SOX9 Promotes Lineage Plasticity and Progression in Basal-like Breast Cancer
Source: Cell Rep. Author manuscript; Available in PMC 2020 Nov 12. (PMC7658810; doi:10.1016/j.celrep.2020.107742)
Supplement: 1 [file NIHMS1641520-supplement-1.pdf]

Cell Reports, Volume *31*

Supplemental Information

Stem Cell Determinant SOX9 Promotes Lineage Plasticity and Progression in Basal-like Breast Cancer

John R. Christin, Chunhui Wang, Chi-Yeh Chung, Yu Liu, Christopher Dravis, Wei Tang, Maja H. Oktay, Geoffrey M. Wahl, and Wenjun Guo

Figure S1

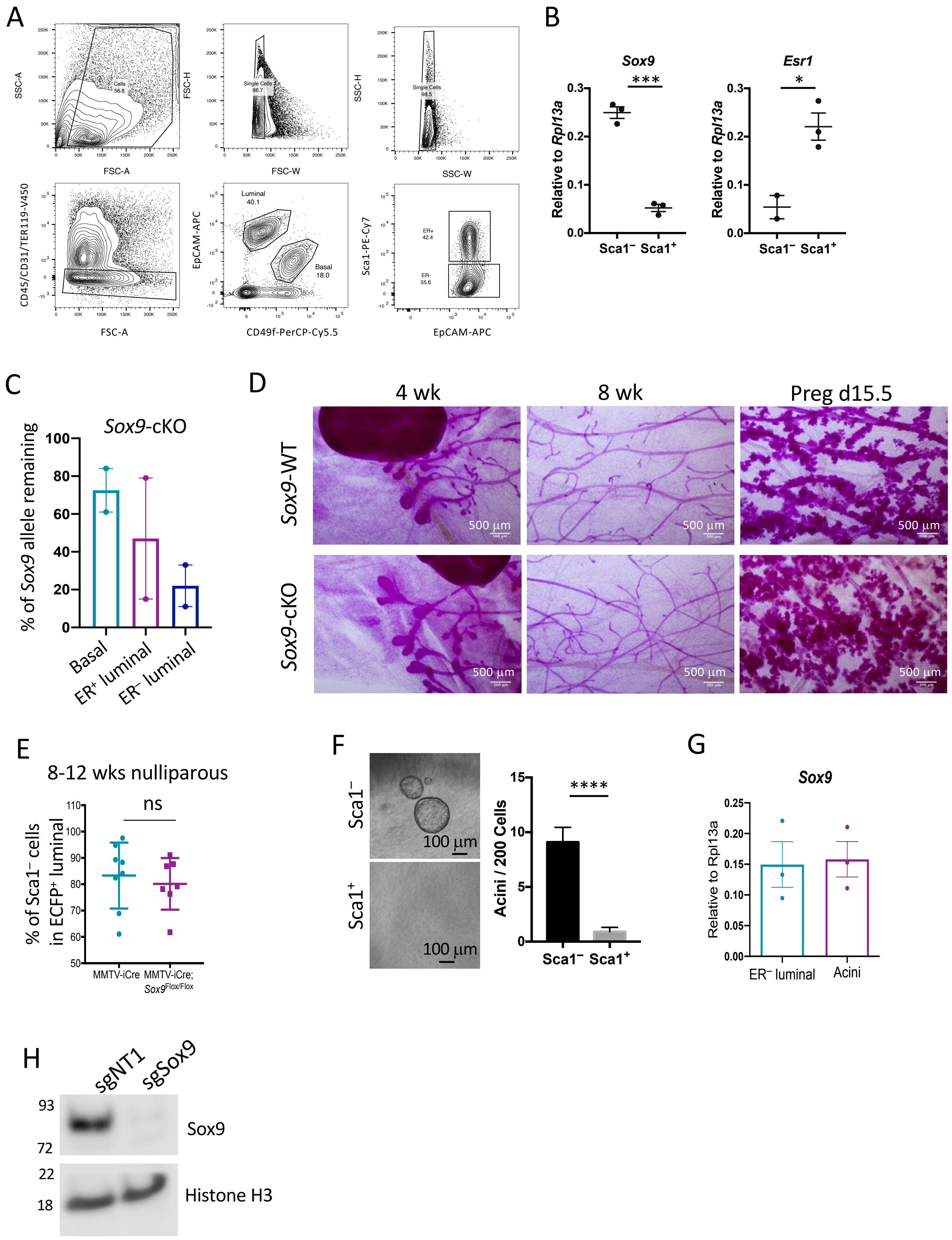
A
B
Sox9
Esr1
Relative to Rpl13a
Sca1⁻ Sca1⁺
C
Sox9-cKO
% of Sox9 allele remaining
Basal
ER⁺ luminal
ER⁻ luminal
D
4 wk
8 wk
Preg d15.5
Sox9-WT
Sox9-cKO
500 µm
E
8-12 wks nulliparous
ns
% of Sca1⁻ cells in ECFP⁺ luminal
MMTV-iCre
MMTV-iCre; Sox9Flox/Flox
F
Sca1⁻
Sca1⁺
100 µm
Acini / 200 Cells
G
Sox9
Relative to Rpl13a
ER⁻ luminal
Acini
H
sgNT1
sgSox9
93
72
22
18
Sox9
Histone H3

Figure S1. ***Sox9*** **deletion** ***in vivo*** **inhibits ER$^-$ luminal stem/progenitor activity, related to Figure 1.**

A. FACS gating strategy for sorting basal, ER$^+$ luminal cells and ER$^-$ luminal cells.
B. Expression levels of *Sox9* and *Esr1* in ER$^-$ and ER$^+$ luminal cells freshly sorted from normal mammary glands of C57BL/6J mice, as measured by qRT-PCR (n = 3 per group). *P < 0.05, ***P < 0.001.
C. *Sox9* allele frequency was measured by qPCR in FACS sorted mammary epithelial populations from ~3 months *Sox9*-cKO mice (n = 2).
D. Wholemount carmine stain of the mammary gland at 4 weeks, 8 weeks, and pregnancy day 15.5.
E. Frequency of ER– cells in ECFP+ luminal cells of animals at 8-12 weeks of age, as measured by flow cytometry (n = 7-8 per group).
F. Organoid-forming ability of ER- and ER+ luminal cells. Cells were sorted from normal mammary glands of C57BL/6J mice and cultured in organoid medium for 7 days. Representative results of three experiments are shown. ****P < 0.0001.
G. *Sox9* expression levels in ER- luminal cell-derived acini and freshly sorted ER- luminal cells, as compared by qRT-PCR (n=3 mice per group)
H. Western blot validation of SOX9 CRISPR knockout in acini.

Figure S2

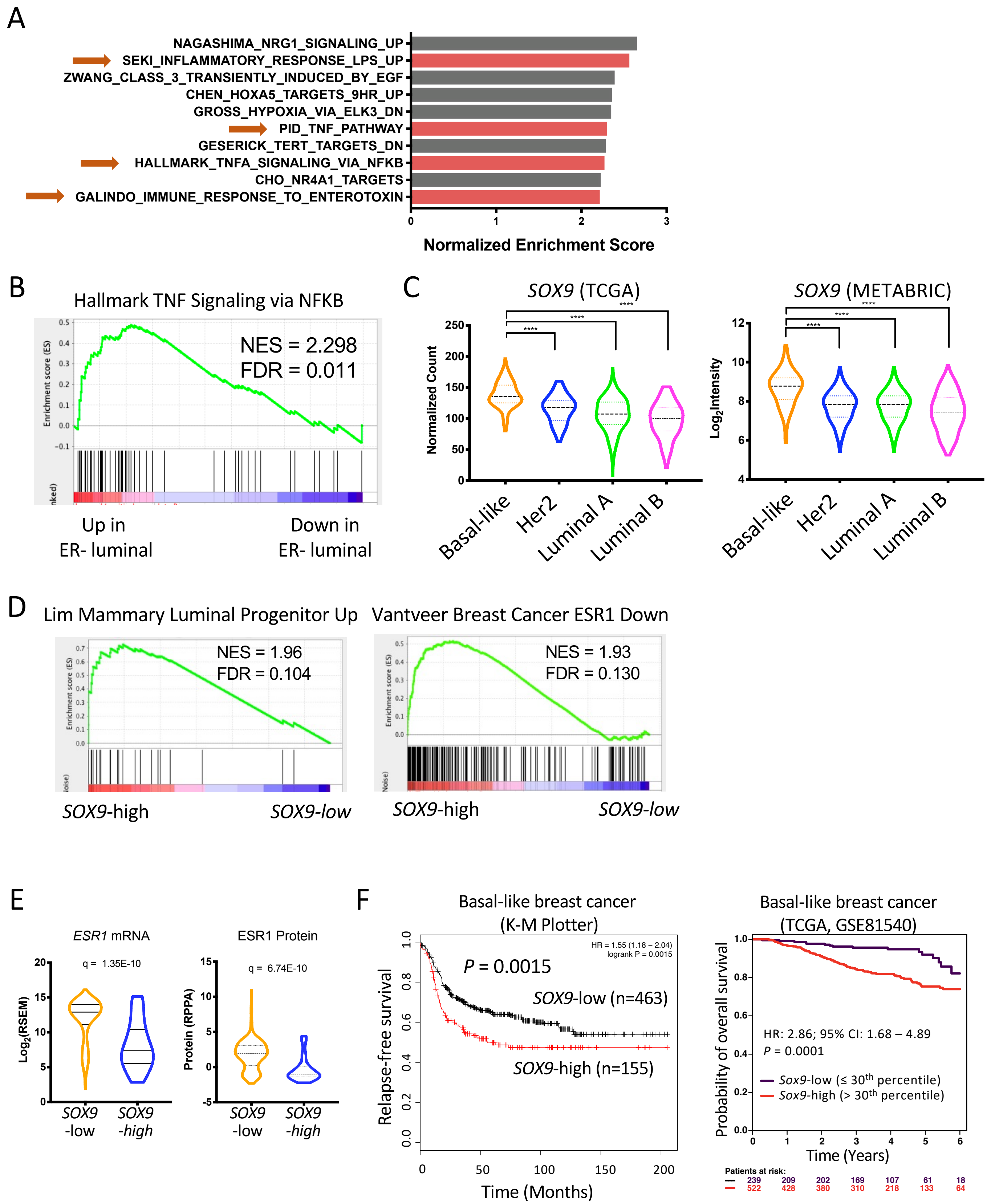

A
NAGASHIMA_NRG1_SIGNALING_UP
SEKI_INFLAMMATORY_RESPONSE_LPS_UP
ZWANG_CLASS_3_TRANSIENTLY_INDUCED_BY_EGF
CHEN_HOXA5_TARGETS_9HR_UP
GROSS_HYPOXIA_VIA_ELK3_DN
PID_TNF_PATHWAY
GESERICK_TERT_TARGETS_DN
HALLMARK_TNFA_SIGNALING_VIA_NFKB
CHO_NR4A1_TARGETS
GALINDO_IMMUNE_RESPONSE_TO_ENTEROTOXIN
Normalized Enrichment Score
B
Hallmark TNF Signaling via NFKB
NES = 2.298
FDR = 0.011
Enrichment score (ES)
Up in ER- luminal
Down in ER- luminal
C
SOX9 (TCGA)
Normalized Count
SOX9 (METABRIC)
Log2Intensity
Basal-like
Her2
Luminal A
Luminal B
D
Lim Mammary Luminal Progenitor Up
NES = 1.96
FDR = 0.104
Vantveer Breast Cancer ESR1 Down
NES = 1.93
FDR = 0.130
SOX9-high
SOX9-low
E
ESR1 mRNA
q = 1.35E-10
Log2(RSEM)
ESR1 Protein
q = 6.74E-10
Protein (RPPA)
SOX9 -low
SOX9 -high
F
Basal-like breast cancer (K-M Plotter)
HR = 1.55 (1.18 – 2.04) logrank P = 0.0015
P = 0.0015
SOX9-low (n=463)
SOX9-high (n=155)
Relapse-free survival
Time (Months)
Basal-like breast cancer (TCGA, GSE81540)
HR: 2.86; 95% CI: 1.68 – 4.89
P = 0.0001
Sox9-low (≤ 30th percentile)
Sox9-high (> 30th percentile)
Probability of overall survival
Time (Years)
Patients at risk:
239 209 202 169 107 61 18
522 428 380 310 218 133 64

Figure S2. *Sox9* deletion leads to NF-κB signaling defect *in vivo*, related to Figure 2.

A. Ten most enriched gene sets in *Sox9*-WT cells compared to *Sox9*-cKO cells. Four of the ten gene sets are associated with NF-κB signaling (arrows).
B. Preranked GSEA of scRNA-seq data showing an enrichment of NF-κB signaling in ER^{-} luminal cells.
C. The expression levels of *SOX9* in various breast cancer subtypes in the TCGA (n = 837) and METABRIC (n = 1110) studies. Statistics were done by Kruskal-Wallis test with Dunn's multiple comparison correction. ****q < 0.0001.
D. Molecular signatures enriched in *SOX9*-high cancer samples in the TCGA RNA-Seq dataset, as determined by GSEA using SOX9 expression as a continuous variable. FDR < 0.25 is considered statistically significant in GSEA.
E. ESR1 protein and *ESR1* mRNA are both significantly downregulated in *SOX9*-high (Z-Score ≥ 2) tumors in the TCGA cohort. SOX9-high n = 68, SOX9-low n = 1025.
F. Higher *SOX9* levels correlate with shorter relapse free survival or overall survival in BLBC. Left: *SOX9*-high and -low BLBC samples in the KM Plotter database were stratified and compared using auto select cut off. Right: *SOX9*-high and -low BLBC samples in the TCGA and GSE81540 datasets were stratified and compared by the optimal setting in bc-GenExMiner. The P value was calculated by log-rank test.

Figure S3

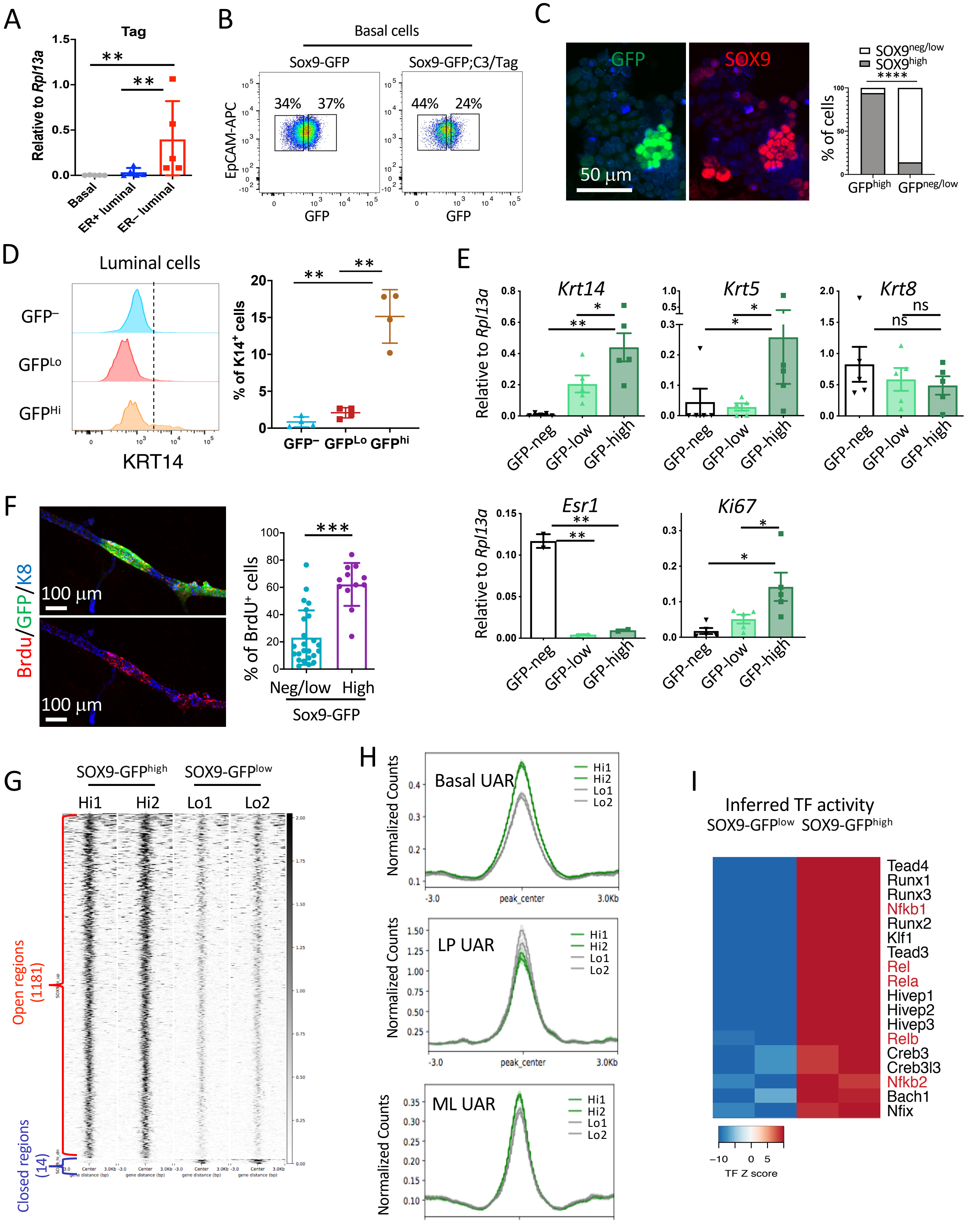

A
Tag
Relative to Rpl13a
Basal
ER+ luminal
ER– luminal
B
Basal cells
Sox9-GFP
Sox9-GFP;C3/Tag
EpCAM-APC
GFP
34%
37%
44%
24%
C
GFP
SOX9
50 µm
SOX9neg/low
SOX9high
% of cells
GFPhigh
GFPneg/low
D
Luminal cells
GFP–
GFPLo
GFPHi
KRT14
% of K14+ cells
GFP– GFPLo GFPhi
E
Krt14
Krt5
Krt8
Relative to Rpl13a
GFP-neg
GFP-low
GFP-high
ns
Esr1
Ki67
F
Brdu/GFP/K8
100 µm
% of BrdU+ cells
Neg/low High
Sox9-GFP
G
SOX9-GFPhigh
SOX9-GFPlow
Hi1 Hi2 Lo1 Lo2
Open regions (1181)
Closed regions (14)
H
Basal UAR
LP UAR
ML UAR
Normalized Counts
peak_center
Hi1
Hi2
Lo1
Lo2
I
Inferred TF activity
SOX9-GFPlow SOX9-GFPhigh
Tead4
Runx1
Runx3
Nfkb1
Runx2
Klf1
Tead3
Rel
Rela
Hivep1
Hivep2
Hivep3
Relb
Creb3
Creb3l3
Nfkb2
Bach1
Nfix
TF Z score

Figure S3. Upregulation of SOX9 during C3(1)/Tag BLBC tumorigenesis results in luminal-to-basal reprogramming, related to Figure 3.

A. Tag expression levels in sorted mammary epithelial populations of 2-3 months old C3(1)/Tag mice (n = 5) were measured by qRT-PCR.
B. Flow cytometric analysis of GFP levels in basal cells from Sox9-GFP or Sox9-GFP;C3(1)/Tag mice at ~3 months old. No significant change was observed between 2 groups.
C. Mammary epithelial cells from 3 months old Sox9-GFP;C3(1)/Tag mice were cytospun, and then immunostained for SOX9. Percentages of SOX9-high or SOX9-neg/low cells in GFP-high (n = 71) or GFP-neg/low cells (n = 663) were quantified.
D. Luminal cells from ~3-month old Sox9-GFP; C3(1)/Tag mice (n = 4) with different levels of GFP were analyzed for KRT14 expression by intracellular flow cytometry. Only Sox9-GFP^{Hi} luminal cells gained the expression of KRT14. Statistics were done by one-way ANOVA with Tukey multiple comparison correction. **$P < 0.01$.
E. The expression levels of basal (*Krt14* and *Krt5*) and luminal (*Krt8*) keratins, *Esr1*, and *Ki67* in luminal cells expressing various levels of Sox9-GFP reporter, as determine by qRT-PCR. n = 5 Sox9-GFP;C3(1)/Tag animals. *$P < 0.05$, **$P < 0.01$.
F. BrdU incorporation of GFP-high or GFP-neg/low luminal cells in ~3-month old Sox9-GFP;C3(1)/Tag mice (n = 2). Mice were injected with BrdU by i.p. (200 μg/mouse). 10 hours later, mammary glands were harvested, cryo-sectioned and stained with anti-BrdU and anti-KRT8 antibodies. A representative image of mammary ducts was shown (left). The graph shows frequencies of $BrdU^+$ cells among GFP-neg/low or GFP-high $KRT8^+$ cells in individual mammary duct sections (n = 12-24 ducts).
G. Waterfall plot showing significantly open or closed chromatin regions comparing Sox9-GFP^{Hi} and Sox9-GFP^{low} luminal cells from ~3-month old Sox9-GFP; C3(1)/Tag mice (n = 2 biological samples, 4 animals per sample).
H. Average ATAC-seq signals of basal, luminal progenitor (LP), and mature luminal (ML) unique accessible regions (UARs) in Sox9-GFP^{Low} or Sox9-GFP^{high} luminal cells from ~3-months Sox9-GFP; C3(1)/Tag mice (n = 2 biological samples, 4 animals per sample).
I. ChromVAR analysis of ATAC-seq data comparing TF activity between Sox9-GFP^{low} and Sox9-GFP^{high} cells. The top TFs with higher activities in Sox9-GFP^{high} cells were shown.

Figure S4

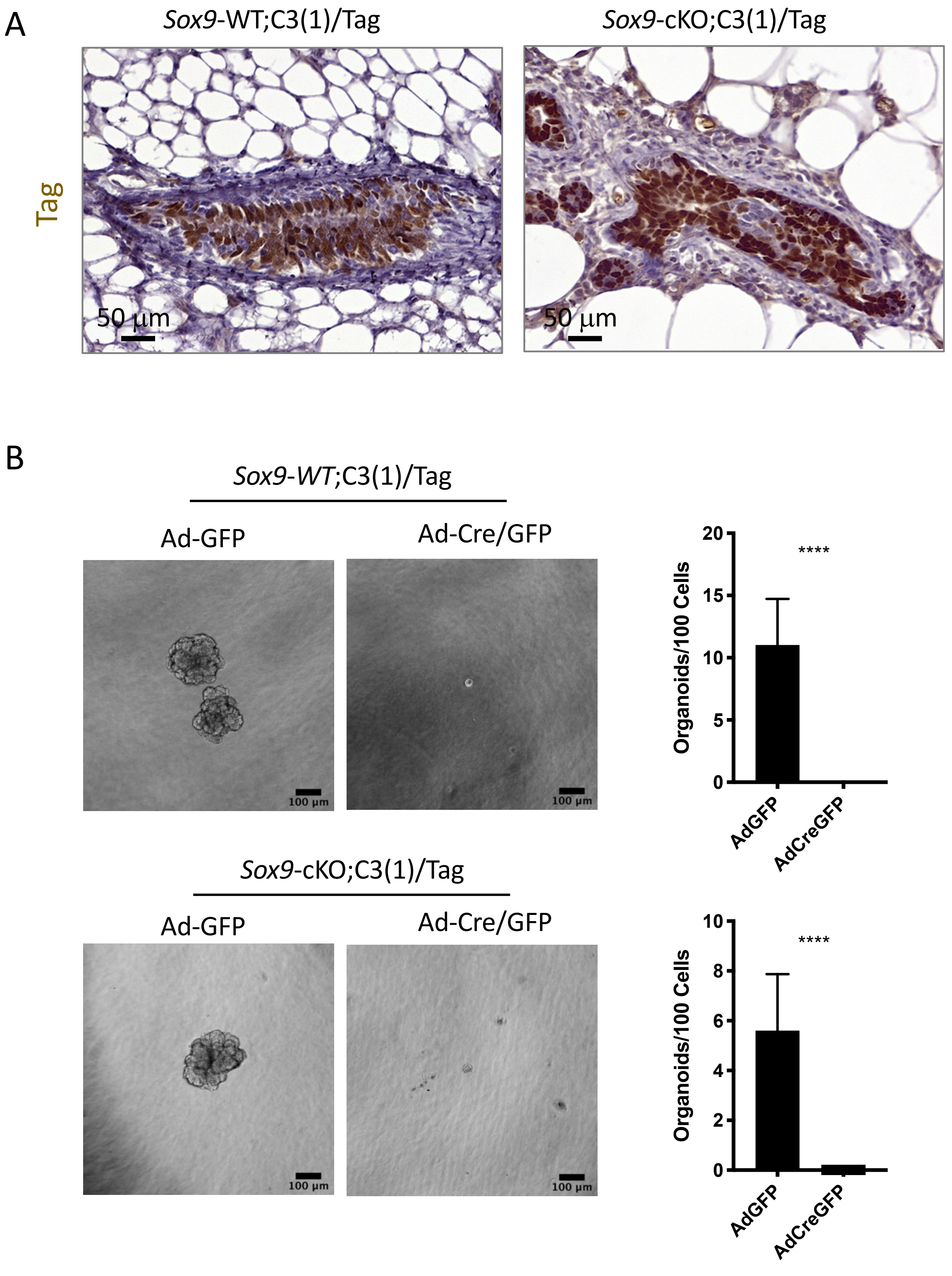
A
Sox9-WT;C3(1)/Tag
Sox9-cKO;C3(1)/Tag
Tag
50 µm
50 µm
B
Sox9-WT;C3(1)/Tag
Ad-GFP
Ad-Cre/GFP
100 µm
100 µm
Organoids/100 Cells
20
15
10
5
0
****
AdGFP
AdCreGFP
Sox9-cKO;C3(1)/Tag
Ad-GFP
Ad-Cre/GFP
100 µm
100 µm
Organoids/100 Cells
10
8
6
4
2
0
****
AdGFP
AdCreGFP

Figure S4. *Sox9* deletion inhibits the progression of benign lesions to invasive carcinoma, related to Figure 4.

A. Expression of the Tag protein in WT (*Sox9*$^{F/F}$;C3(1)/Tag) and Sxo9-cKO (MMTV-iCre;*Sox9*$^{F/F}$;C3(1)/Tag) MINs, as determined by immunohistochemistry.
B. Freshly isolated tumor cells from *Sox9*-WT;C3(1)/Tag or *Sox9*-cKO;C3(1)/Tag tumor were infected with adenovirus expressing either Cre/GFP or GFP. The cells were then measured for their organoid-forming ability. Representative results from 2 experiments were shown. ****P < 0.0001.

Table S1. Genotyping primers, Related to STAR Methods.

Primer Name	Genotype	Primer Sequence
11576	$Sox9^{Flox}$	AGACTCTGGGCAAGCTCTGG
11577	$Sox9^{Flox}$	GTCATATTCACGCCCCCATT
Sox9-floxed1	$Sox9^{WT}$	GGGGCTTGTCTCCTTCAGAG
Sox9-floxed3	$Sox9^{WT}$	TGGTAATGAGTCATACACAGTAC
oIMR8543	$Sox9^{WT}$<br>Internal Positive Control	GGTTAAACCCAGCTTGACCA
oIMR8544	$Sox9^{WT}$<br>Internal Positive Control	GGAGGCAGAGACAGTTGGAG
iCre-new-F	MMTV-iCre	CTCTGACAGATGCCAGGACA
iCre-new-R	MMTV-iCre	TCTCTGCCCAGAGTCATCCT
Slug-YFP-3	MMTV-iCre<br>Internal Positive Control	GGGAAAAGCCTTTCTCTTGCCCTC
Slug-YFP-4	MMTV-iCre<br>Internal Positive Control	GGTTGGTAAGCACATGAGAAAATGTC
oIMR0069	C3(1)-TAg	GGACAAACCACAACTAGAATGCAGTG
oIMR0068	C3(1)-TAg	CAGAGCAGAATTGTGGAGTGG
oIMR8745	C3(1)-TAg<br>Internal Positive Control	GTCAGTCGAGTGCACAGTTT
oIMR8744	C3(1)-TAg<br>Internal Positive Control	CAAATGTTGCTTGTCTGGTG

Table S2. RT-PCR primers, Related to STAR Methods.

Primer Name	Primer Sequence
mSox9RT F	AGGAAGCTGGCAGACCAGTA
mSox9RT R	CGTTCTTCACCGACTTCCTC
mESR1 F	CGTGTGCAATGACTATGCCTC
mESR1 R	TTTCATCATGCCCACTTCGTAA
mElf5 F	ATGTTGGACTCCGTAACCCAT
mElf5 R	GCAGGGTAGTAGTCTTCATTGCT
mID4-new F	AGGGTGACAGCATTCTCTGC
mID4-new R	CCGGTGGCTTGTTTCTCTTA
h/m Sox10 F	CCCACACTACACCGACCAG
h/m Sox10 R	GGCCATAATAGGGTCCTGAGG
Tnfaip3 FWD	CCACTTGGGCTCTGCGAGG
Tnfaip3 REV	TTCTGGGGTTCTCTCTCGTATCT
Tnfaip2 FWD	TTAAGATCGAGGTGGCCACA
Tnfaip2 REV	CACTGCTTGGTAGATTGCCCT
Tnf FWD	ATGGCCTCCCTCTCATCAGT
Tnf REV	TTTGCTACGACGTGGGCTAC
Rel FWD	GCTCTGCCTCCCATTGTTTC
Rel REV	AGTTCTTGTTCACACGGCAGA
Rela FWD	CTGTGCCTACCCGAAACTCA
Rela REV	AGGGATGCTGGGAAGGTGTA
Relb FWD	GTCACTAACGGTCTCCAGGA
Relb REV	GCCAAAGCCGTTCTCCTTAAT
Nfkb1 FWD	GGAGTCACGAAATCCAACGC
Nfkb1 REV	CCTCGTCATCACTCTTGGCA
Nfkb2 FWD	TCCTTCGTAGTTACAAGCTGGC
mKeratin 5 F	TCTGCCATCACCCCATCTGT
mKeratin 5 R	CCTCCGCCAGAACTGTAGGA
mKeratin 8 F	TCCATCAGGGTGACTCAGAAA
mKeratin 8 R	CCAGCTTCAAGGGGCTCAA
Krt14 FWD	GCGGCCCACTGAGATCAAA
Krt14 REV	TCGATCTGCAGGAGGACATTG
Rpl13a FWD	CGAAGATGGCGGAGGGG
Rpl13a REV	CCTTCACAGCGTACGACCAC

Table S3. Cloning primers, Related to STAR Methods.

Primer Name	Sequence
sgSox9 Exon 1	GTACCCGCATCTGCACAACG
sgNfkb2 Exon 2	TCCTTAGGCTCCACGATGGA
sgNfkb2 Exon 5	CTGTGGGCATGCGCACGAGG
sgNT1	GCGAGGTATTCGGCTCCGCG
Nfkbia Mut FWD	GATCTCGAGCTCAAGCTTCGATGTTTCAGCCAGCTGGG
Nfkbia Mut REV	AGAATTATCTAGAGTCGCGGTTATAATGTCAGACGCTGGC
